# Integrated analysis of single-cell RNA sequencing, transcriptomics, and thermal proteome profiling identifies PLCG1 as the therapeutic target of isopimpinellin in treating rheumatoid arthritis

**DOI:** 10.1186/s11658-026-00918-8

**Published:** 2026-04-04

**Authors:** Huantian Cui, Ning Wang, Feitian Min, Huan Pei, Yuming Wang, Hanzhou Li, Qianqian Wan, Yan Meng, Mingwei He, Xiaoman Lv, Liwei Xing, Zixuan Li, Tianze Pan, Renlin Li, Weibo Wen, Xiangying Kong, Yuhong Bian

**Affiliations:** 1 First School of Clinical Medicine, Yunnan University of Chinese Medicine, Kunming, 650500 China; 2 School of Integrative Chinese and Western Medicine, Tianjin University of Traditional Chinese Medicine, Tianjin, 301617 China; 3 Department of Rheumatology and Immunology, The First Affiliated Hospital of Xinjiang Medical University, Urumchi, 830054 China; 4 Science and Technology Department, Yunnan University of Chinese Medicine, Kunming, 650500 China; 5 Medical School, Xizang University, Lhasa, 850000 China; 6 Second School of Clinical Medicine, Yunnan University of Chinese Medicine, Kunming, 650500 China; 7 Institute of Chinese Materia Medica, China Academy of Chinese Medical Sciences, Beijing, 100700 China

**Keywords:** Isopimpinellin, Rheumatoid arthritis, Macrophage polarization, SPP1, PLCG1, Fibroblast-like synoviocytes, scRNA-seq

## Abstract

**Background:**

Isopimpinellin (ISOP), derived from *Toddalia asiatica* (L.) Lam, is thought to possess anti-inflammatory potential. However, the effects of ISOP on rheumatoid arthritis (RA) and corresponding mechanisms remain unclear.

**Methods:**

We evaluated the therapeutic effect of ISOP on RA using the collagen-induced arthritis (CIA) rat model. Subsequently, we determined the potential mechanisms of ISOP by combining single-cell RNA sequencing of rat synovial tissue in vivo with transcriptome analysis of macrophages in vitro. Molecular docking, thermal proteome profiling, cellular thermal shift assays, and drug affinity responsive target stability were then employed to identify the molecular targets of ISOP. Finally, we validated this target and explored the interaction between macrophages and RA-fibroblast-like synoviocytes (FLS) in vitro.

**Results:**

We found that ISOP improved pathological changes in CIA rats. Mechanistically, ISOP inhibited macrophage migration and M1 macrophage polarization, and downregulated *Spp1* expression. Furthermore, ISOP restrained the RAS/ERK pathway in M1 macrophages. Importantly, PLCG1 is a direct target of ISOP. Silencing PLCG1 reduced the related inhibitory effects of ISOP on M1 macrophage polarization. In addition, the supernatant of macrophages treated with ISOP reduced the proliferation and activation of RA-FLS. Silencing PLCG1 eliminated the regulatory effects of ISOP in the macrophage-RA-FLS co-culture system.

**Conclusions:**

ISOP exerts its anti-RA effects by targeting PLCG1 to inhibit the production of SPP1 in M1 macrophages.

**Supplementary Information:**

The online version contains supplementary material available at 10.1186/s11658-026-00918-8.

## Introduction

Rheumatoid arthritis (RA) is primarily characterized by joint swelling, pain, and dysfunction and is a chronic, recurrent systemic inflammatory disease. Pathological features of RA include synovitis, articular cartilage destruction, and bone erosion [1]. Among these, synovitis—often recurring—is a key factor driving disease progression [2]. Synovitis involves a variety of cell types, including immune cells (macrophages, dendritic cells, and lymphocytes) and nonimmune cells (fibroblast-like synoviocytes (FLS) and vascular endothelial cells). The activation of immune cells produces inflammatory mediators that promote FLS proliferation and activation. In turn, proliferating and activated FLS release factors such as extracellular matrix components that further activate immune cells. This cycle exacerbates articular cartilage destruction and bone erosion, leading to recurrent disease flare-ups and progression [3]. RA treatments currently include traditional disease-modifying antirheumatic drugs, nonsteroidal anti-inflammatory drugs, and biologics. However, these therapies often face challenges such as disease recurrence following discontinuation [4, 5]. Therefore, controlling synovitis is essential for effective RA management.

The anti-inflammatory effects of natural products have been well established. Numerous studies have demonstrated that natural products, such as berberine and celastrol, exert anti-RA activity by inhibiting synovitis [6, 7]. Identifying new natural products and elucidating their mechanisms of action are critical for advancing RA treatment. However, most current research on the anti-RA mechanisms of natural products focuses on individual cell types. The complexity of synovitis, which involves multiple cell types, makes it insufficient to study single cells alone in order to fully understand the anti-RA mechanisms of natural products. Single-cell RNA sequencing (scRNA-seq) enables a deeper exploration of changes in the immune microenvironment during RA synovitis by examining single-cell heterogeneity and intercellular interactions. Studies using scRNA-seq have identified two fibroblast subsets, DAF^+^ and THY1^+^, in the joints of patients with RA, predominantly located in the synovial lining [8]. These subsets display invasive proliferation and apoptosis resistance, characteristic of RA onset and progression, and secrete large amounts of inflammatory mediators, contributing to inflammatory hyperplasia in the RA synovium [9]. Additionally, scRNA-seq has shown that macrophages in the sub-synovial layer are the primary drivers of inflammation [10], further amplifying FLS aggressiveness by releasing inflammatory cytokines, such as IL-1β, which promote FLS-mediated joint damage [11]. Targeting macrophage polarization and inhibiting the abnormal proliferation of FLS may be key therapeutic strategies for RA [12]. Thus, combining scRNA-seq technology allows for a multicellular perspective on the anti-RA mechanisms of natural products, enabling the identification of direct target cells and molecules involved in their action.

*Toddalia asiatica* (L.) Lam, commonly known as Feilong Zhangxue, is a medicinal plant native to southwestern China and other regions, with a long history of use in treating RA. Previous studies have confirmed the therapeutic effects of *Toddalia asiatica* (L.) Lam extracts in RA animal models [13]. However, the active compounds and underlying mechanisms of its anti-RA effects remain unclear. Isopimpinellin (ISOP), a major bioactive ingredient of *Toddalia asiatica* (L.) Lam [14], exhibits anti-inflammatory, anti-tumor, and antioxidant properties and may be the key compound responsible for its anti-RA activity [15–17]. In this study, we first assessed the anti-RA effects of ISOP using the collagen-induced arthritis (CIA) rat model. Next, we applied scRNA-seq to explore the transcriptional landscape of synovial tissue in RA rats and investigate the potential mechanisms underlying ISOP’s anti-RA effects. We also established an in vitro M1 macrophage model and combined transcriptome analysis, Transwell assays, molecular docking, thermal proteome profiling (TPP), cellular thermal shift assays (CETSA), and drug affinity responsive target stability (DARTS) techniques to identify and validate ISOP’s targets. Furthermore, we evaluated the effects of ISOP on RA-FLS proliferation and activation using a co-culture system of M1 macrophages and RA-FLS. This study not only clarifies the multilayered anti-RA mechanisms of ISOP, providing valuable insights for its targeted application, but also identifies, for the first time, the pathway through which M1 macrophages produce SPP1. These findings contribute to a deeper understanding of the regulatory mechanisms of SPP1 in RA.

## Materials and methods

### Reagents

**Fig. 1:**
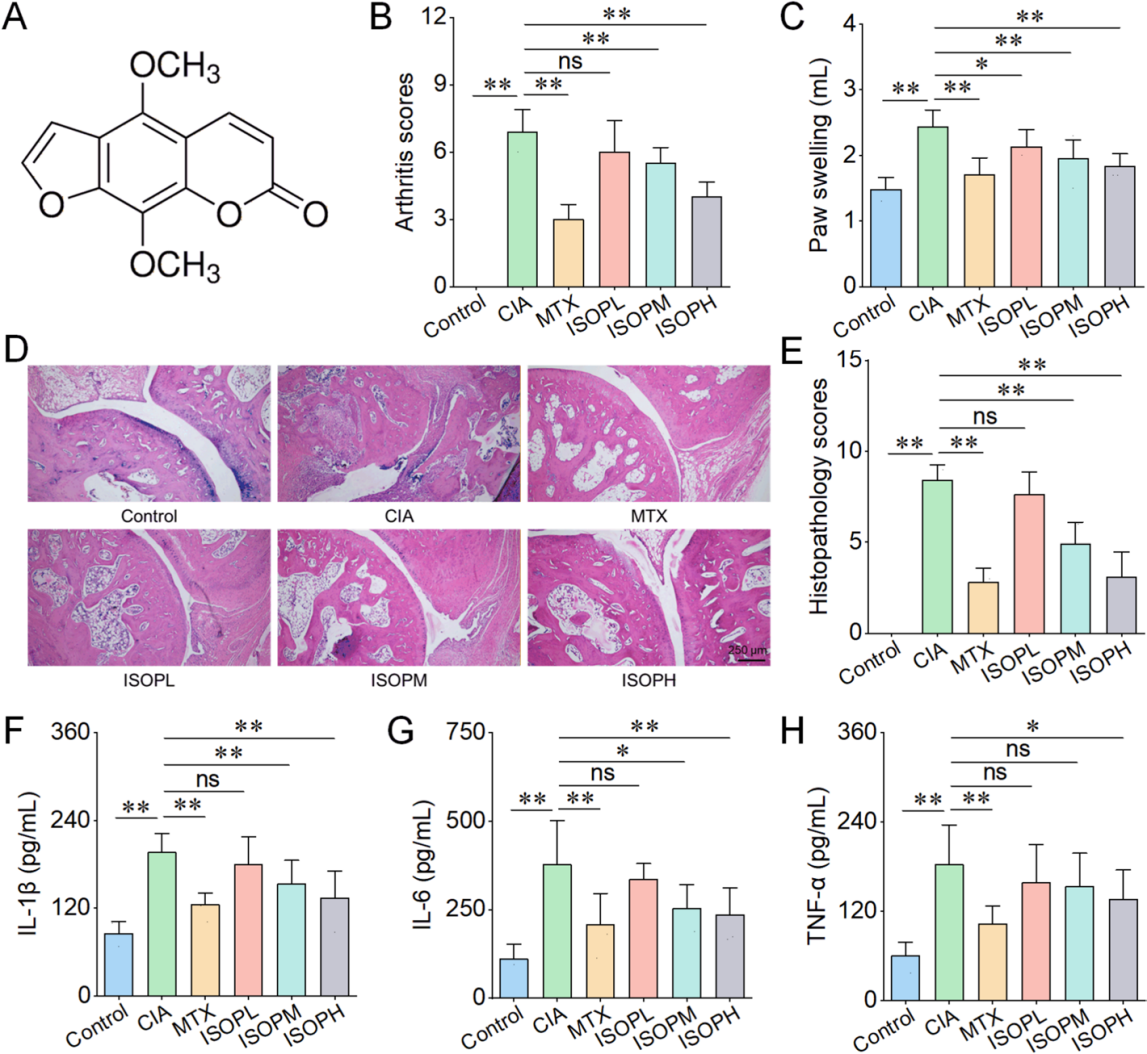
ISOP ameliorates joint pathological damage and inflammatory infiltration in CIA rats. **a** The structural formula of ISOP. **b**, **c** ISOP intervention improves the arthritis index (**b**) and paw swelling (**c**) in CIA rats. **d, e** ISOP intervention alleviates pathological symptoms (**a**) and reduces ankle joint pathology scores (**e**) in CIA rats. **f**–**h** ISOP intervention decreases serum levels of IL-1β (**f**), IL-6 (**g**), and TNF-α (**h**) in CIA rats. Data presented as mean ± SD. *n* = 10 per group. *P < 0.05; **P < 0.01; ns, not significant

The molecular formula of ISOP is C_13_H_10_O_5_, with a purity of ≥ 98%. Its structural formula is shown in Fig. 1a. Detailed information on the reagents used in this study is provided in the Supplementary Information.

### In vivo experiments

#### Animals

We purchased specified pathogen-free (SPF)-grade male Sprague Dawley (SD) rats aged 6–8 weeks and weighing 200 ± 20 g from Beijing HFK Bioscience Co., Ltd. (experimental animal license no. SCXK (Jing) 2020-0004). The rats were housed five per cage in a room maintained at 20–24 °C with 50–60% relative humidity, on a 12 h light/dark cycle, and given free access to food and water. This study involving animals was approved by the Experimental Ethics Review Committee of Yunnan University of Chinese Medicine (ethical approval no. YNUCM-XMSB-G-20250281) and was performed following the Guidelines for the Care and Use of Animals of the Experimental Animal Management Committee (Yunnan, China). The committees adhere to the ethical standards of the International Council for Laboratory Animal Science (ICLAS).

#### Modeling, grouping, and drug administration

After 7 days of adaptive feeding, 10 rats were randomly selected from the 60 rats to form the Control group using a random number table. The remaining rats were used to establish the CIA animal model according to previously described methods [18]. Briefly, incomplete Freund’s adjuvant (IFA) was mixed with bovine type II collagen (CII) solution in equal volumes to form a CII-containing emulsion (2 mg/mL). Rats in the modeling group received a subcutaneous injection of the collagen emulsion at the base of the tail (0.2 mL, day 0, and 0.1 mL, day 7), while rats in the normal group received an equal volume of vehicle at the same site. Arthritis severity was assessed using an arthritis index. The CIA model was considered to be established 21 days after the first injection, and the corresponding scores are shown in Additional file 1: Fig. S1.

After modeling, the 50 rats were randomly assigned to five groups: the model group (CIA), the methotrexate (MTX) group, the low-dose ISOP group (ISOPL), the medium-dose ISOP group (ISOPM), and the high-dose ISOP group (ISOPH), with 10 rats per group. Drug intervention began on day 21 and continued once daily for 28 days. Rats in the MTX group received daily intraperitoneal injections of 0.5 mg/kg MTX, while rats in the ISOPL, ISOPM, and ISOPH groups were given orally of 25, 50, and 100 mg/kg/day, respectively. Rats in the normal and CIA groups received daily intraperitoneal injections of an equal volume of saline. On day 49, rats were anesthetized with 50 mg/kg pentobarbital sodium, and their arthritis index and paw swelling were measured. Rats were then euthanized to collect blood samples and bilateral hind ankle joints.

#### Paw swelling and arthritis index

Volume of the rat paws was measured using a plethysmometer. Paw swelling was calculated as the difference in paw volume before and after modeling. Arthritis index scoring criteria were based on previous study [19], with detailed information provided in the Supplementary Information.

#### Histopathological staining

We fixed rat hind ankle joints in a 4% paraformaldehyde solution for 48 h, and subsequently decalcified, dehydrated, cleared, and embedded with paraffin. Longitudinal sections, 3 μm thick, were prepared from the joints. Select tissue sections were stained with hematoxylin and eosin (HE) and examined under a microscope to assess histopathological changes in the ankle joints. Pathological evaluation was performed based on established criteria from previous study [19], with detailed information provided in the Supplementary Information.

#### Single-cell RNA sequencing

We processed rat synovial tissue to generate a single-cell suspension. We labeled cells with barcodes and constructed a library. Sequencing was performed using Illumina NovaSeq PE150 with quality-checked raw data using fastp. A cell-gene expression matrix was generated with Cell Ranger 7.1.0. Cells were filtered on the basis of metrics such as the number of detected genes and mitochondrial UMI proportion. DoubletFinder was applied to predict and remove doublets, eliminating multicellular contaminants from the scRNA-seq data. Using the filtered cell-gene expression matrix, we standardized the data with the LogNormalize function in Seurat 4.3.0. Principal components were selected through PCA, and clustering was refined using a graph-based method. The *t*-SNE dimensionality reduction algorithm visualized cell spatial distribution in two dimensions. We calculated and analyzed differentially expressed genes (DEGs) and their fold change (FC) among clusters using the FindMarkers function. Batch effects were corrected in multisample integration analyses with Seurat CCA.

Marker genes were identified using the Wilcoxon rank sum test, defining each cluster. We then conducted intragroup proportion analyses and inter-group DEG analyses for the defined clusters. Gene Ontology (GO)/Kyoto Encyclopedia of Genes and Genomes (KEGG) enrichment analyses of marker genes or DEGs were performed with clusterProfiler 3.14.0.

### In vitro experiments

#### Culture and induction of THP-1 cells

THP-1 cells (#SCSP-567, Cell Bank of the Chinese Academy of Sciences, China) were cultured in RPMI 1640 medium supplemented with 10% fetal bovine serum (FBS), 0.05 mM β-mercaptoethanol, and 1% penicillin–streptomycin (P/S) at 37 °C in a 5% CO_2_ incubator with saturated humidity. Logarithmic growth phase cells were used for all experiments. To induce differentiation into macrophages, PMA (100 ng/mL) was added to the complete medium, and the cells were incubated for 24 h. Subsequently, M1 polarization was achieved by treating the cells with LPS (100 ng/mL) and IFN-γ (20 ng/mL) for 24 h. SPP1 is a secretory protein; to allow the detection of SPP1 in cell lysate, we treated THP-1 cells with BFA (1 μg/mL) for 3 h after M1 polarization to inhibit SPP1 secretion.

#### Culture and induction of MH7A cells

The RA-FLS cell line MH7A (#SCSP-5528, Cell Bank of the Chinese Academy of Sciences, China) was cultured in DMEM complete medium supplemented with 10% FBS and 1% (P/S) at 37 °C in a 5% CO_2_ incubator with saturated humidity. Cells in the logarithmic growth phase of MH7A were selected for subculturing and subsequent experiments.

#### MTT assay for cell viability

We seeded THP-1 cells and MH7A cells into 96-well plates at a density of 1 × 10^4^ cells per well and treated with varying concentrations of ISOP. After a 24-h incubation at 37 °C in 5% CO_2_, we added MTT solution to each well. Following an additional 4-h incubation, we removed supernatant and added 150 μL of dimethyl sulfoxide (DMSO) to each well. Low-speed oscillation dissolved the resulting crystals, and absorbance at 490 nm was measured using a microplate reader.

### Silencing or overexpression of PLCG1 in THP-1 cells

For PLCG1 silencing, THP-1 cells were seeded into 24-well plates at 4 × 10^4^ cells per well and differentiated into macrophages using PMA. We then replaced the medium with medium containing the Lipo3000 transfection reagent, shPLCG1, and shNC, and then incubated for 6 h. Afterward, the medium was exchanged for one without the transfection reagent or shRNA to prepare the cells for subsequent interventions. The sense strand of shPLCG1 is GGAAGACCTTCCAGGTCAA, and the antisense strand of shPLCG1 is TTGACCTGGAAGGTCTTCC.

To overexpress PLCG1, we obtained the human PLCG1 coding sequence from NCBI and constructed the pcDNA3.1-PLCG1 overexpression vector, which was transformed into plasmids. THP-1 cells were seeded into 24-well plates at 4 × 10^4^ cells/well and differentiated into macrophages using PMA. The medium was then replaced with medium containing the Lipo3000 transfection reagent, oePLCG1 plasmid (pcDNA3.1-PLCG1), or the empty plasmid (pcDNA3.1), and the cells were incubated for 6 h. Afterward, the medium was replaced with one lacking the transfection reagent and plasmid for subsequent experiments.

#### Transwell assay

PMA-differentiated THP-1 cells were seeded into the lower chamber of a Transwell system. LPS and IFN-γ were added to induce M1 polarization, along with drug interventions for 24 h. Subsequently, PMA-differentiated THP-1 cells were seeded into the upper chamber of the Transwell system. After a 24-h incubation, we collected the supernatant to measure inflammatory cytokine levels. We then removed the upper chamber, and cells adhering to its inner surface were gently scraped off. The remaining cells were fixed with 4% paraformaldehyde for 10 min and stained with 0.1% Crystal Violet for 20 min. Staining results were examined under a microscope. Five fields of view at 200× magnification were randomly selected for cell counting.

#### Transcriptomics

Cells with or without ISOP intervention were collected for transcriptomic analysis, following methods described in previous studies [20]. Total RNA was extracted, and its purity, concentration, and integrity were assessed to ensure sample quality. RNA samples meeting the quality standards were used for library preparation and sequencing on the Illumina platform. DESeq2 software was employed to identify DEGs between the M1+ ISOP− versus M1− ISOP− groups and the M1+ ISOP+ versus M1+ ISOP− groups, applying a screening threshold of |log_2_FC|> 0.5 and *P*-value < 0.05. Enrichment analyses, including GO and KEGG, were performed on the identified DEGs to explore functional and pathway implications.

#### Cytokine level detection

Serum from rats or cell supernatants from each experimental group was collected. The levels of IL-1β, IL-6, and TNF-α were measured using ELISA kits.

#### Immunofluorescence

In vivo experiments involved dewaxing pathological sections, performing antigen retrieval, and incubating the sections with 3% H_2_O_2_ solution at room temperature. The sections were then blocked with 20% goat serum for 1 h. In vitro experiments, after completing cellular interventions, included fixing cells with 4% paraformaldehyde for 15 min at room temperature. The cells were permeabilized using 0.5% Triton X-100 and blocked with goat serum for 30 min. Following washing, both sections and cells were incubated overnight at 4 °C with primary antibodies targeting the protein of interest. After three washes, we added and incubated fluorescent secondary antibodies at room temperature for 1 h. DAPI staining was used to visualize cell nuclei. The samples were mounted with an antifade mounting medium and analyzed using a confocal laser scanning microscope. Positive expression areas were quantified using Image Pro Plus 6.0 software.

#### Western blot

We extracted total protein from synovial tissue or cells and determined protein concentrations using the BCA method. Proteins were separated by SDS-PAGE electrophoresis. We then transferred them to a PVDF membrane using the wet transfer method. The membranes were blocked with 5% skimmed milk for 2 h and incubated overnight at 4 °C with primary antibodies targeting the protein of interest. After three washes with TBST, membranes were incubated with HRP-conjugated secondary antibodies for 2 h at room temperature. Following another three washes, excess liquid was removed, and ECL reagent was applied for detection. Immunoblot bands were visualized using an automatic gel imaging system. Grayscale values of the protein bands were quantified using ImageJ software for statistical analysis.

#### RT-qPCR

Using a total RNA extraction kit, we extracted total RNA from synovial tissue and cells, and conducted reverse transcription to synthesize cDNA. The relative mRNA expression levels of target genes were determined using qPCR and normalized to Actb expression. Relative expression levels were calculated using the 2^−ΔΔCT^ method. The forward primer of *SPP1* is AGCAGCTTTACAACAAATACCCAG, and the reverse primer of *SPP1* is TTACTTGGAAGGGTCTGTGGG. The forward primer of *ACTB* is AGCCTTCCTTCCTGGGCAT, and the reverse primer of *ACTB* is CTTCATTGTGCTGGGTGCC.

#### Molecular docking

The molecular docking method followed protocols established in previous studies [21]. The 2D chemical structure of ISOP was obtained from the PubChem database (https://pubchem.ncbi.nlm.nih.gov/) and saved in SDF format. The 3D structure of the PLCG1 protein was retrieved from the PDB database (https://www.rcsb.org/) and saved in PDB format. Using PyMOL software, we removed water molecules and small-molecule ligands from the protein structure. Hydrogen atoms were added to the structures using AutoDock Tools software to prepare the receptor and ligand for docking. Molecular docking was performed to assess the binding ability of ISOP to PLCG1, with an *S* score < −5.0 kcal/mol considered indicative of strong binding. Docking results were visualized to analyze binding interactions and spatial conformation.

#### TPP and CETSA

The TPP methodology followed protocols described in previous studies [22–24]. In brief, THP-1 cells were differentiated into M1 macrophages using PMA, LPS, and IFN-γ. The cells were then collected and resuspended in PBS containing 1% protease and phosphatase inhibitors at a concentration of 5 × 10^5^ cells/mL. We subjected them to four cycles of freeze–thaw (30 s in liquid nitrogen followed by 30 s at 37 °C). The lysate was centrifuged at 4 °C for 25 min at 12,000×*g*, and the supernatant was collected. The supernatant was divided into six tubes: three were treated with 100 μM ISOP and three with an equal volume of DMSO. The samples were incubated at 37 °C for 1 h, followed by heating at 60 °C for 5 min and rapid cooling on ice. After centrifugation at 4 °C for 25 min at 12,000×*g*, equal volumes of supernatant were collected for subsequent proteomic analysis.

For the CETSA, we followed methods outlined in the literature [22, 23]. The protein supernatant obtained from the freeze–thaw process in the TPP experiment was divided into 18 tubes: 9 were treated with 100 μM ISOP and the remaining 9 with an equal volume of DMSO. The samples were incubated at 37 °C for 1 h, followed by exposure to temperatures ranging from 37 to 68 °C (in 4 °C increments) for 5 min. The samples were rapidly cooled on ice. After centrifugation at 4 °C for 25 min at 12,000×*g*, we collected equal volumes of supernatant, and performed Western blot analysis to detect the expression levels of PLCG1. Finally, CETSA melting curves were plotted at different temperatures to assess the thermal stability of proteins.

#### DARTS

We used DARTS to validate the binding of ISOP to the PLCG1 protein, following methods described in previous studies [25–27]. Briefly, the cell protein supernatant obtained through repeated freeze–thaw cycles, as outlined in the TPP protocol, was mixed with TNC solution (10×) at a 9:1 ratio (protein supernatant:TNC). Protein concentration was quantified using the BCA method, and the samples were divided into six tubes. Three tubes were treated with 100 μM ISOP, while the other three received an equal volume of DMSO, and all samples were incubated at 37 °C for 1 h. Next, varying concentrations of pronase E were added to the DMSO and ISOP groups at pronase E to protein mass ratios of 1/500, 1/1000 and 1/2000. A Control group without pronase E intervention was also included. We incubated samples at room temperature for 30 min, followed by Western blot analysis to detect PLCG1 expression.

#### Statistical analysis

We performed statistical analysis using SPSS Pro online. Data are presented as mean ± SD, and normality was evaluated using the Shapiro–Wilk test. Differences between groups were assessed using Student’s unpaired *t*-test, one-way ANOVA, or two-way ANOVA, as appropriate. When unequal variances were detected, Welch’s correction was applied. A *P*-value < 0.05 was considered statistically significant.

## Results

### ISOP exhibits therapeutic effects on CIA rats

Rats in the CIA group exhibited significantly higher arthritis index and paw swelling compared with the Control group. Histological analysis with HE staining revealed several pathological changes in the joint tissues of the CIA group, including narrowed joint spaces, abnormal proliferation of lining cells and fibroblasts in the synovial tissue, extensive inflammatory cell infiltration, and increased ankle joint pathology scores. Both MTX and ISOP treatments improved the arthritis index and reduced paw swelling in CIA rats to varying extents (Fig. 1b, c). Furthermore, the MTX and ISOP interventions alleviated pathological changes in the ankle joints of CIA rats, leading to a reduction in ankle joint pathology scores (Fig. 1d, e). In addition, serum levels of IL-1β, IL-6, and TNF-α were significantly elevated in the CIA group compared with the Control group. Treatment with MTX and ISOP reduced the serum levels of these inflammatory cytokines in the CIA rats to varying degrees (Fig. 1f–h). Notably, the high-dose ISOP treatment did not show a significant difference in improving synovial tissue and ankle joint pathology compared with MTX. On the basis of these findings, we selected high-dose ISOP for further studies.

### ISOP intervention inhibits M1 macrophage polarization and downregulates SPP1 expression

**Fig. 2:**
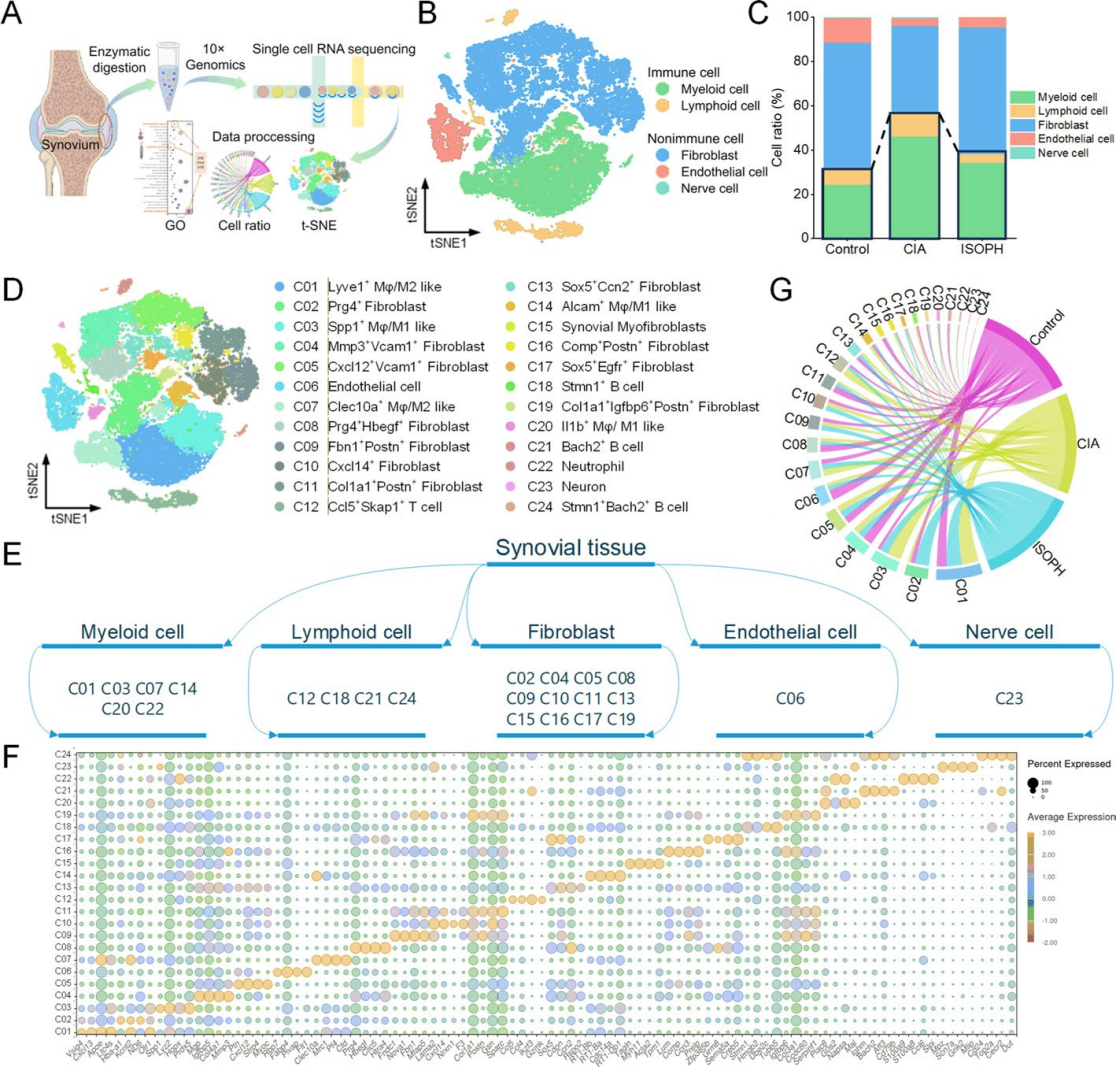
Analysis of macrophages based on scRNA-seq. **a** Schematic diagram of the scRNA-seq analysis workflow. **b** Integrated results and preliminary cell definitions of scRNA-seq from synovial tissues of Control, CIA, and ISOPH groups based on *t*-SNE. Cells are defined as myeloid, lymphoid, fibroblast, endothelial, and nerve. **c** Relative proportions of the five cell types in each group. **d** Further definitions of the five cell types grouped into 24 clusters based on *t*-SNE. **e** Diagram showing the relationship between the five cell types and the 24 clusters. **f** Bubble heatmap showing the relationship between the 24 clusters and marker genes. **g** Relative proportions of the 24 clusters in each group

We selected three rats from each group (Control, CIA, and ISOPH), isolated synovial tissue from the same location, and performed scRNA-seq to analyze the effects of ISOP on cell types in the synovial tissue of CIA rats (Fig. 2a). The scRNA-seq data from the three groups were integrated for cell type analysis. *t*-SNE analysis classified the cells into five major categories: myeloid cell, lymphoid cell, fibroblast, endothelial cell, and nerve cell (Fig. 2b). Among these, myeloid and lymphoid cells are immune cells, while fibroblast, endothelial cell, and nerve cell are nonimmune cells. The expression distribution of each marker gene in the *t*-SNE plot is shown in Additional file 1: Fig. S2. The relative proportions of each cell type in the three groups are depicted in Fig. 2c. Notably, myeloid and lymphoid cells, both immune cell types, predominated across all groups, with increased immune cell infiltration in the synovium of the CIA group. This infiltration decreased following ISOP treatment. Further *t*-SNE dimensionality reduction identified 24 distinct clusters (Fig. 2d). The relationship between these clusters and the five major cell types is shown in Fig. 2e. The heatmap of marker gene expression for each cluster is presented in Fig. 2f, while the *t*-SNE expression distribution of marker genes for each cluster is shown in Additional file 1: Fig. S3. These results confirm that the marker genes for each population are highly expressed within their respective clusters. Additionally, the intragroup percentages of the 24 clusters are displayed in Fig. 2g. ISOP treatment significantly altered the proportions of various immune cell subsets, including C01, C03, C07, C12, C14, C18, C20, C21, C22, and C24. We next focused on analyzing the effects of ISOP intervention on myeloid and lymphoid cells.

**Fig. 3:**
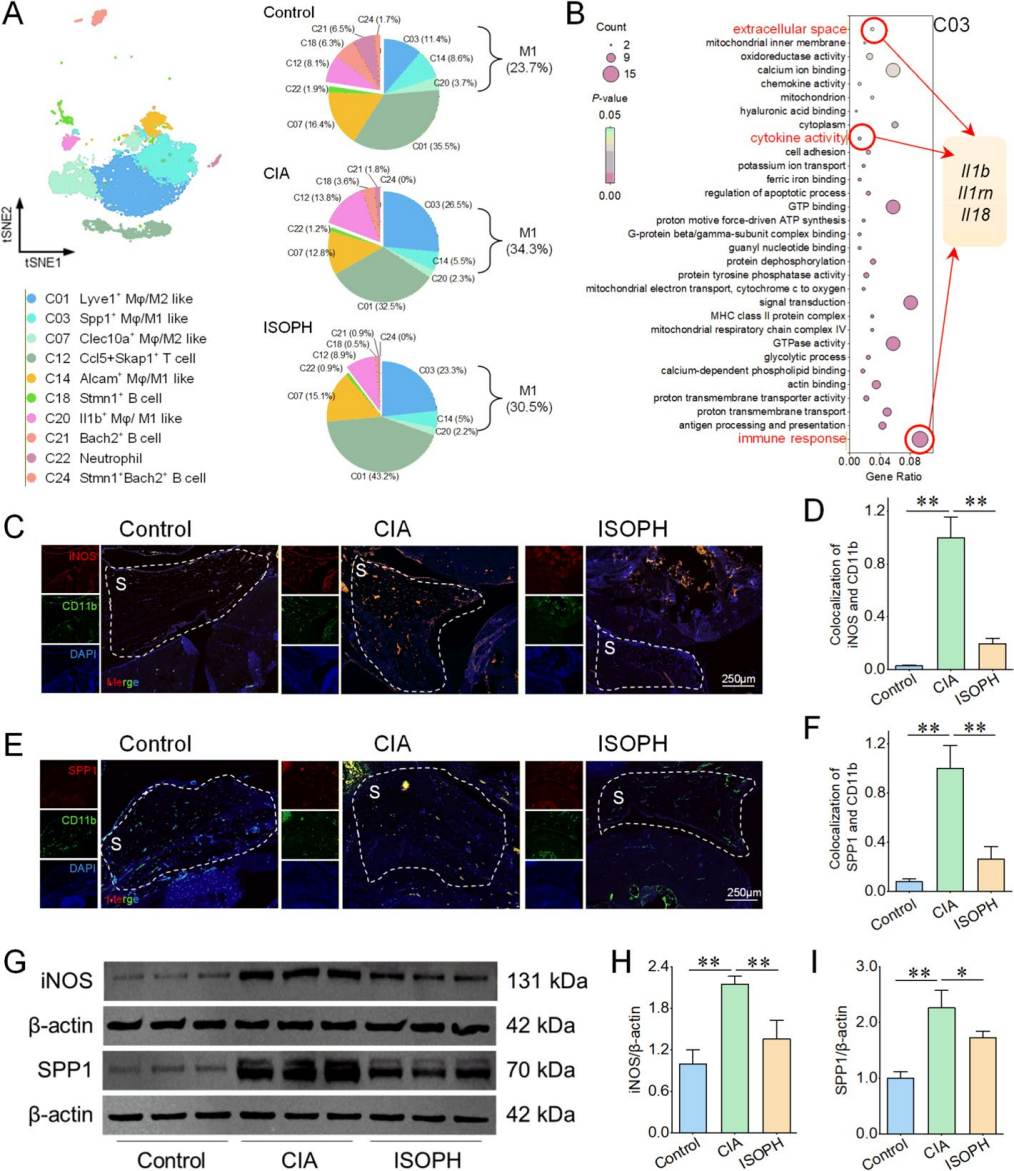
ISOP intervention inhibits M1 macrophage polarization. **a** Relative proportions of immune cell subsets in Control, CIA, and ISOPH groups. ISOP intervention reverses the increase in the relative proportion of M1. **b** GO analysis of marker genes for C03, proinflammatory genes *Il1b*, *Il1rn*, and *Il18*, are enriched in the pathways for immune response, extracellular space, and cytokine activity. **c–f** Immunofluorescence results show that ISOP intervention reduces the area of iNOS^+^ CD11b^+^ (**c**, **d**) and SPP1^+^ CD11b^+^ (**e**, **f**) positive regions in the synovial membrane (marked as “S” in figures) of CIA rats. **g**–**i** Western blot results show that ISOP intervention downregulates iNOS (**g**, **h**) and SPP1 (**g**, **i**) protein expression. *n* = 3 per group

As shown in Fig. 3a, we examined the relative proportions of each immune cell (myeloid and lymphoid cells) subset in the Control, CIA, and ISOPH groups. We found that M1-like macrophages (C03, C14, C20) and M2-like macrophages (C01, C07) were the predominant immune cell populations. ISOP intervention significantly reduced the proportion of M1-like macrophages, particularly C03 (*Spp1*^+^ macrophages). Given the key role of M1 macrophages in synovial inflammation in RA, we performed GO enrichment analysis on the marker genes of each M1-like macrophages subset. The results showed that the marker genes of the C03 population were significantly enriched in pathways related to immune response, extracellular space, and cytokine activity. Pro-inflammatory genes such as *Il1b*, *Il1rn*, and *Il18* were notably enriched in these pathways (Fig. 3b). Similarly, the GO enrichment results for the marker genes of C14 and C20 suggested that these populations primarily promote inflammation (Additional file 1: Fig. S4). Immunofluorescence analysis revealed that, compared with the Control group, the area of M1 (iNOS^+^ CD11b^+^) positive regions in the joint synovium of CIA rats significantly increased. However, following ISOP treatment, the area of M1 positive regions decreased substantially (Fig. 3c, d). Similarly, ISOP intervention reduced the area of SPP1^+^ CD11b^+^ positive regions (Fig. 3e, f). Western blot analysis confirmed that ISOP treatment downregulated the expression of iNOS and SPP1 proteins (Fig. 3g–i). These results suggest that ISOP may inhibit M1 macrophage polarization, particularly in *Spp1*^+^ macrophages.

### ISOP inhibits M1 macrophage migration and polarization by blocking RAS/ERK-mediated SPP1 expression

**Fig. 4:**
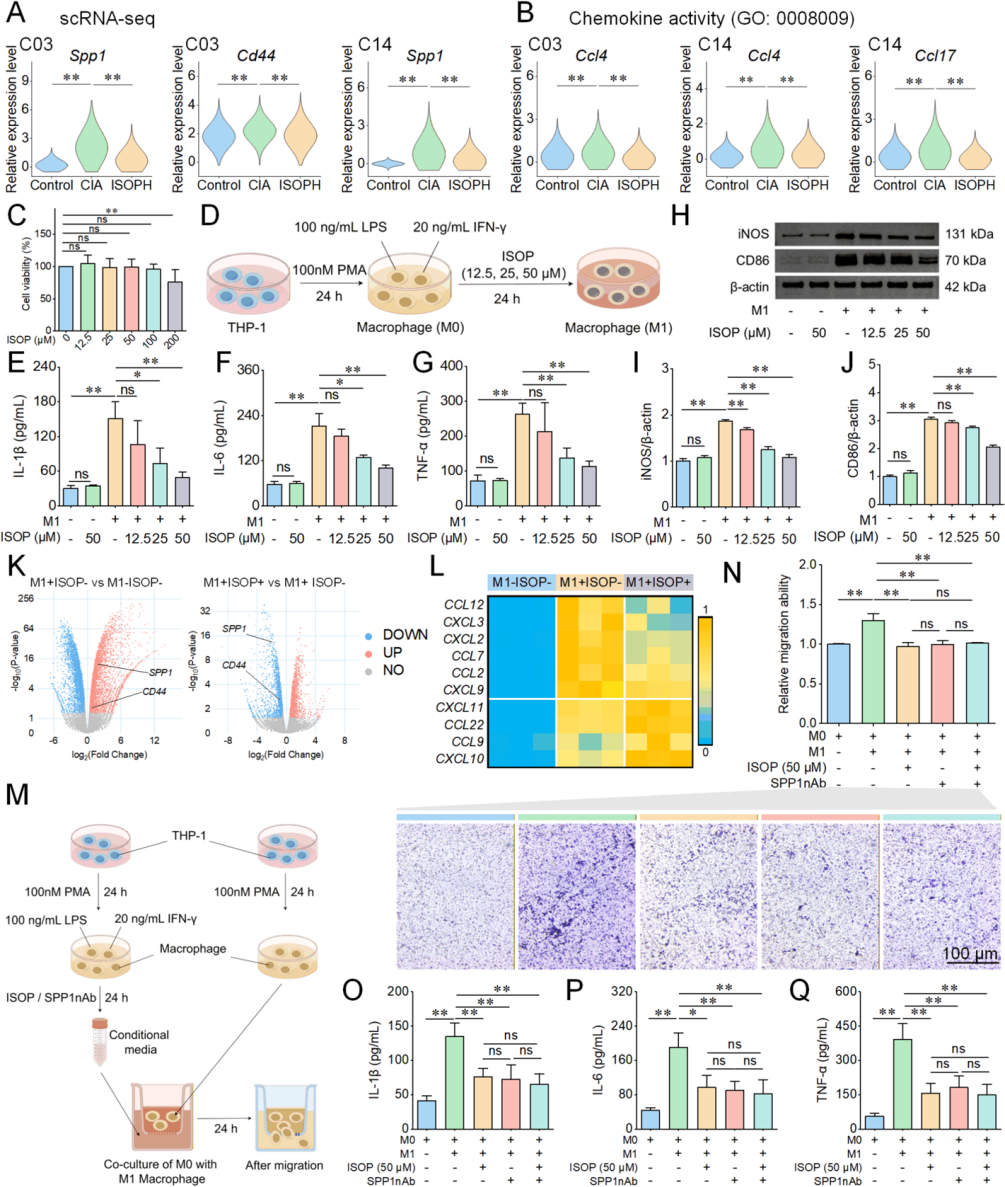
ISOP downregulates SPP1 expression to inhibit macrophage migration and M1 polarization. **a**, **b** scRNA-seq results showing ISOP intervention downregulates *Spp1* and *Cd44* in C03, and *Spp1* expression in C14 (**a**). In chemokine activity (GO: 0008009), ISOP downregulates *Ccl4* in C03, and *Ccl4* and *Ccl17* gene expression in C14 (**b**). **c–k** Subsequently, we explored the in vitro mechanism of ISOP intervention in macrophages. First, we used MTT to screen doses of ISOP without toxic effects on THP-1 cells. Then, we induced differentiation of THP-1 cells into macrophages with 100 nM PMA for 24 h, followed by induction of M1 macrophage polarization with 100 ng/mL LPS and 20 ng/mL IFN-γ for 24 h. Afterwards, we used 12.5, 25, and 50 μM ISOP intervention in THP-1 cells or M1 macrophages to assess effects of ISOP on M1. We also performed transcriptomic analysis on M1 macrophages with and without ISOP intervention to explore the mechanism of ISOP inhibition in M1 macrophage polarization. Finally, we established a Transwell coculture system for THP-1 (M0) and M1 macrophages, applied ISOP intervention and SPP1 neutralizing antibody (SPP1nAb) simultaneously, and assessed the inhibitory effect of ISOP on SPP1-mediated macrophage migration and activation. **c** MTT results showing ISOP below 100 μM had no significant effects on THP-1 cell viability. **d** Schematic diagram of the in vitro experiments. **e–g** ISOP intervention reduces IL-1β (**e**), IL-6 (**f**), and TNF-α (**g**) levels in supernatants. **h–j** ISOP intervention downregulates iNOS (**h, i**) and CD86 (**h, j**) protein expression. **k** Transcriptome gene volcano plot revealing ISOP intervention downregulates *SPP1* and *CD44* expression. **l** ISOP intervention downregulates expression of *CCL12*, *CXCL3*, *CXCL2*, *CCL7*, *CCL2*, and *CXCL9* genes that related to cell migration. **m** Schematic diagram of the experiment of Transwell. **n** Both ISOP and SPP1nAb interventions reduces number of migrating macrophages. **o–q** Both ISOP and SPP1nAb interventions decreases IL-1β (**o**), IL-6 (**p**), and TNF-α (**q**) levels in supernatants of the coculture system. *n* = 3 per group

We next analyzed the effects of ISOP intervention on gene expression in M1 macrophages. Our results showed that ISOP treatment downregulated the expression of *Spp1* and *Cd44* in C03 macrophages, and *Spp1* expression in C14 macrophages (Fig. 4a). Literature reports indicate that the binding of SPP1 and CD44 promotes macrophage migration [28]. Therefore, we examined the differential expression of chemokine activity-related genes (GO ID:0008009) in each subset of M1 macrophages. We found that ISOP downregulated *Ccl4* in C03, and *Ccl4* and *Ccl17* in C14 (Fig. 4b). These findings suggest that ISOP may inhibit macrophage migration.

To further investigate the effects of ISOP on M1 macrophage migration and polarization, we conducted in vitro experiments. First, we screened nontoxic doses of ISOP for THP-1 cells. MTT assays revealed that ISOP treatment below 100 μM had no significant effect on the viability of THP-1 cells (Fig. 4c). On the basis of these results, we used doses of 12.5, 25, and 50 μM for subsequent experiments to evaluate the impact of ISOP on M1 macrophage polarization. We induced THP-1 cells to differentiate into M1 macrophages using LPS combined with IFN-γ, while applying different doses of ISOP (Fig. 4d). The results demonstrated that ISOP treatment dose-dependently reduced the levels of IL-1β, IL-6, and TNF-α in the supernatants of THP-1 cells polarized into M1 macrophages (Fig. 4e–g), and downregulated the expression of iNOS and CD86 in these cells (Fig. 4h–j). This initial finding corroborated the inhibitory effect of ISOP intervention on M1 macrophage polarization. Subsequent transcriptomic analysis revealed that ISOP treatment downregulated the expression of *SPP1* and *CD44*, as well as genes associated with cell migration, including *CCL12*, *CXCL3*, *CXCL2*, *CCL7*, *CCL2*, and *CXCL9* (Fig. 4k, l). These results were consistent with the scRNA-seq data. M1 macrophages secrete numerous chemokines that promote the migration and activation of other macrophages. On the basis of these findings, we hypothesized that ISOP may inhibit macrophage migration and activation by suppressing SPP1. To test this hypothesis, we established a Transwell co-culture system of THP-1 and M1 macrophages and treated the system simultaneously with ISOP and an SPP1 neutralizing antibody (SPP1nAb) to investigate whether ISOP could inhibit SPP1-mediated macrophage migration and activation (Fig. 4m). The results showed that ISOP treatment reduced the number of migrating macrophages (Fig. 4n), and decreased the levels of IL-1β, IL-6, and TNF-α in the supernatants (Fig. 4o–q). These effects were similar to those observed with SPP1nAb, with no significant difference in the outcomes between the two interventions. Notably, intervention with ISOP alone and ISOP combined with SPP1nAb showed no significant difference. This finding further confirmed that ISOP inhibits M1 macrophage migration and polarization by suppressing SPP1 production.

**Fig. 5:**
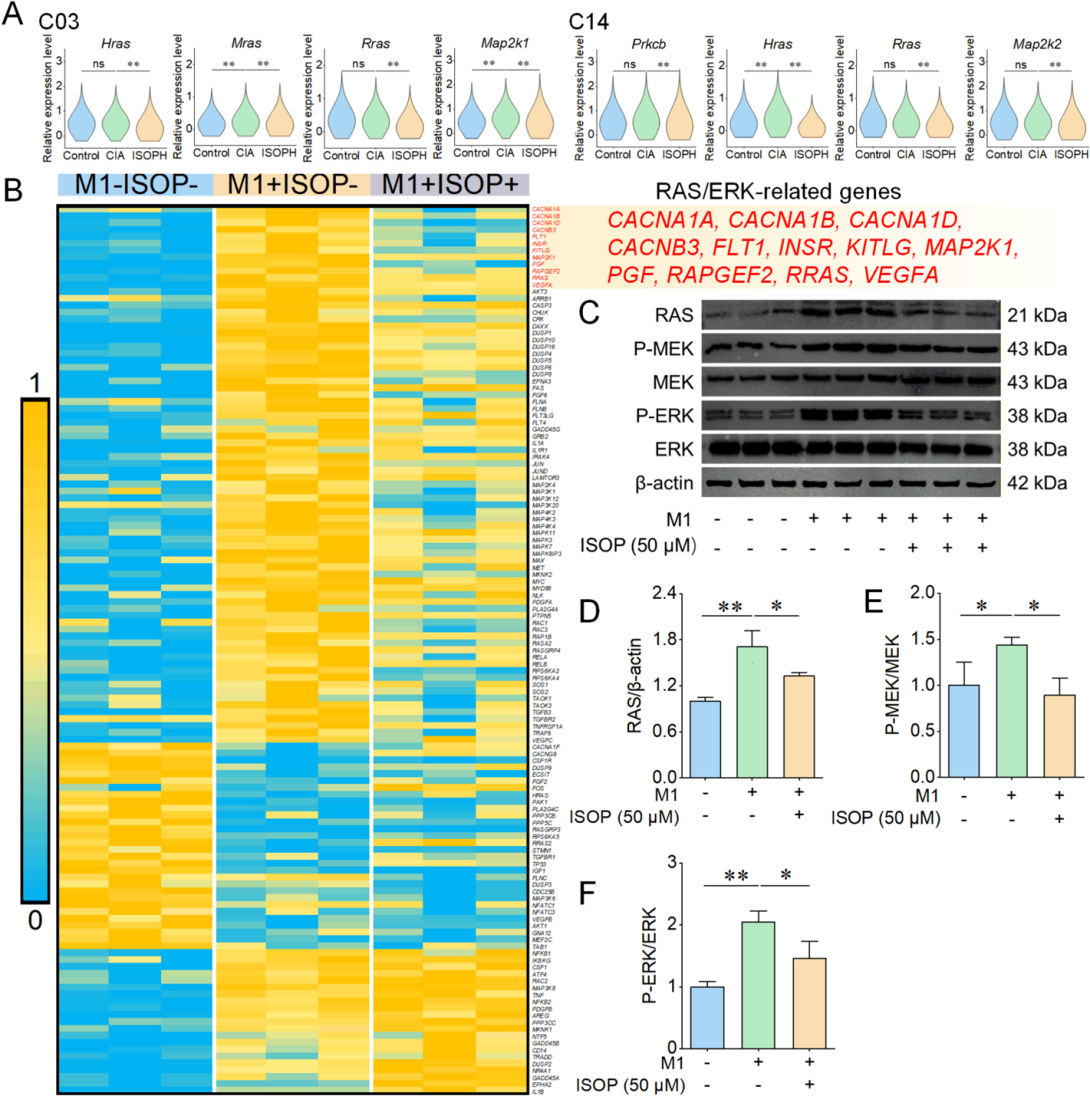
ISOP downregulates SPP1 expression by inhibiting the RAS/ERK pathway. We first evaluated the distribution of RAS/ERK-related genes in C03 and C14. Subsequently, in THP-1 transcriptomic analysis in vitro, we focused on the expression of RAS/ERK-related genes in the MAPK signaling pathway (KEGG ID: map04010) and validated key proteins in the RAS/ERK pathway using Western blot. **a** ISOP intervention downregulates RAS/ERK-related gene expression in C03 and C14. **b** ISOP intervention downregulates expression of most RAS/ERK-related genes in M1 macrophages in the MAPK signaling pathway. **c**–**f** Western blot results showing ISOP intervention reduces expression of RAS (**c**, **d**), P-MEK/MEK (**c**, **e**), and P-ERK/ERK (**c**, **f**) in M1 macrophages. *n* = 3 per group

Previous studies have reported that ERK plays a crucial role in regulating SPP1 production [29], and ERK is a key component of the MAPK signaling pathway. To investigate the involvement of this pathway, we analyzed DEGs related to MAPK signaling (KEGG ID: map04010) in our scRNA-seq data. Results revealed that ISOP intervention downregulated the expression of genes associated with ERK and its upstream regulator, RAS. Specifically, genes such as *Hras*, *Mras*, *Rras*, and *Map2k1* were downregulated in C03, and *Prkcb*, *Hras*, *Rras*, and *Map2k2* showed decreased expression in C14 after ISOP intervention (Fig. 5a). In parallel, in vitro transcriptomic analysis of THP-1 cells indicated that ISOP treatment downregulated the expression of several RAS/ERK-related genes in MAPK signaling pathway, including *CACNA1A*, *CACNA1B*, *CACNA1D*, *CACNB3*, *FLT1*, *INSR*, *KITLG*, *MAP2K1*, *PGF*, *RAPGEF2*, *RRAS*, and *VEGFA* (Fig. 5b). These findings suggest that ISOP may inhibit the RAS/ERK signaling pathway. To further explore this, we treated M1 macrophages with ISOP and used Western blotting to assess the levels of key proteins in the RAS/ERK pathway. The results showed that ISOP intervention reduces expression of RAS, P-MEK/MEK, and P-ERK/ERK (Fig. 5c–f).

### ISOP directly targets PLCG1

**Fig. 6:**
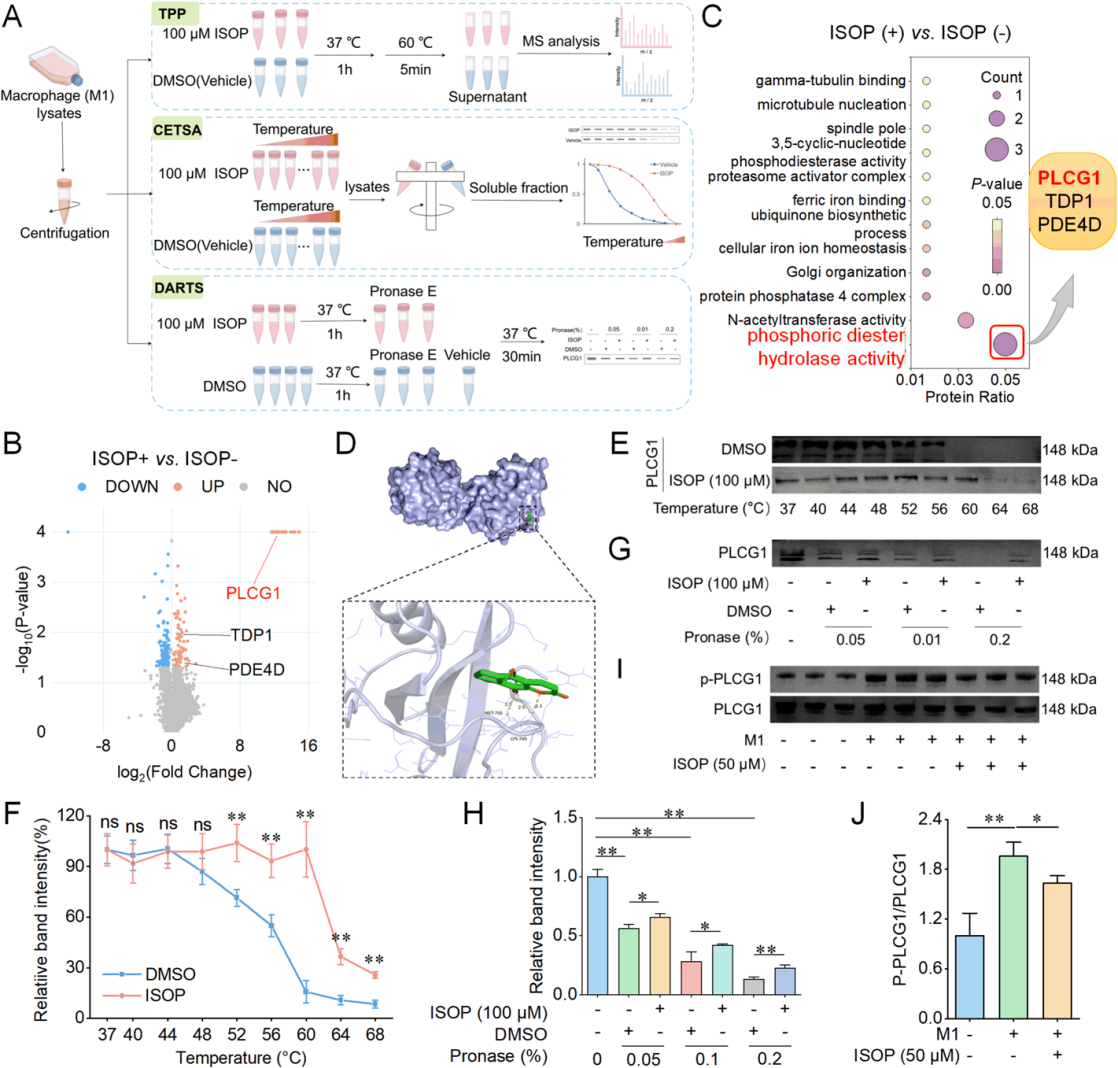
ISOP can directly target PLCG1. **a** Schematic diagram of the TPP, CETAS, and DARTS experiments. **b** Volcano plot depicting differentially expressed proteins screened with FC > 1.2 or < 0.8 and *P*-value < 0.05. PLCG1 is specially marked. **c** GO enrichment results of differentially expressed proteins showing phosphoric diester hydrolase activity as the most significantly enriched. **d** Molecular docking results showing a strong binding capacity between ISOP and PLCG1. **e, f** CETSA results showing ISOP significantly increases thermal stability of PLCG1. **g, h** DARTS results showing ISOP intervention enhances PLCG1 resistance to protease hydrolysis. **i, j** Western blot results showing ISOP intervention reduces PLCG1 phosphorylation levels. *n* = 3 per group

We next used TPP to identify potential targets of ISOP and validated these findings with CETSA, DARTS, and molecular docking (Fig. 6a). Differentially expressed proteins after ISOP treatment were screened based on the criteria of FC > 1.2 or < 0.8 and *P*-value < 0.05 (Fig. 6b). GO enrichment analysis was performed on the proteins with FC > 1.2, and significantly enriched pathways (*P*-value < 0.05) were visualized as a bubble heatmap (Fig. 6c). The results revealed that phosphoric diester hydrolase activity was the most significantly enriched pathway, with PDE4D, TDP1, and PLCG1 identified as key proteins associated with this pathway. Among them, PLCG1 exhibited the largest FC (Fig. 6b). Notably, PLCG1 is known to be a RAS activator [30], which led us to hypothesize that ISOP may inhibit RAS/ERK-mediated SPP1 expression by targeting and binding to PLCG1. Molecular docking results between ISOP and PLCG1 showed a binding energy of −5.6 kcal/mol (< −5 kcal/mol), indicating a high binding affinity and strong interaction between the two molecules. In the docking complex, we observed hydrogen-bond interactions between key amino acid residues, such as Met750 and Lys749, and ISOP. The hydrogen bond distance between Met750 and ISOP was 2.5 pm, and the distance between Lys749 and ISOP was 2.3 pm. These short hydrogen bond distances further confirmed the stability of the ISOP–PLCG1 complex (Fig. 6d). We next used CETSA experiments to confirm the thermostabilizing effect of ISOP on PLCG1. The results showed that, as the temperature increased, the intensity of the specific PLCG1 band gradually decreased. However, after ISOP treatment, the level of undegraded PLCG1 protein increased at the same temperature, and the melting curve of PLCG1 shifted significantly to the right (Fig. 6e, f). This suggests a potential interaction between ISOP and PLCG1, with ISOP enhancing the thermostability of PLCG1. To further validate the effect of ISOP on PLCG1, we conducted DARTS experiments. The results demonstrated that, after ISOP treatment, PLCG1 protein was more resistant to protease hydrolysis (Fig. 6g, h), which was consistent with the CETSA findings. We also treated LPS and IFN-γ-induced M1 macrophages with ISOP and measured the cellular levels of P-PLCG1/PLCG1 by Western blot. The results revealed that ISOP reduced the phosphorylation level of PLCG1 (Fig. 6i, j), indicating that ISOP directly targets PLCG1 and inhibits its activation.

### PLCG1 induces SPP1 production in M1 macrophages by activating the RAS/ERK pathway

**Fig. 7:**
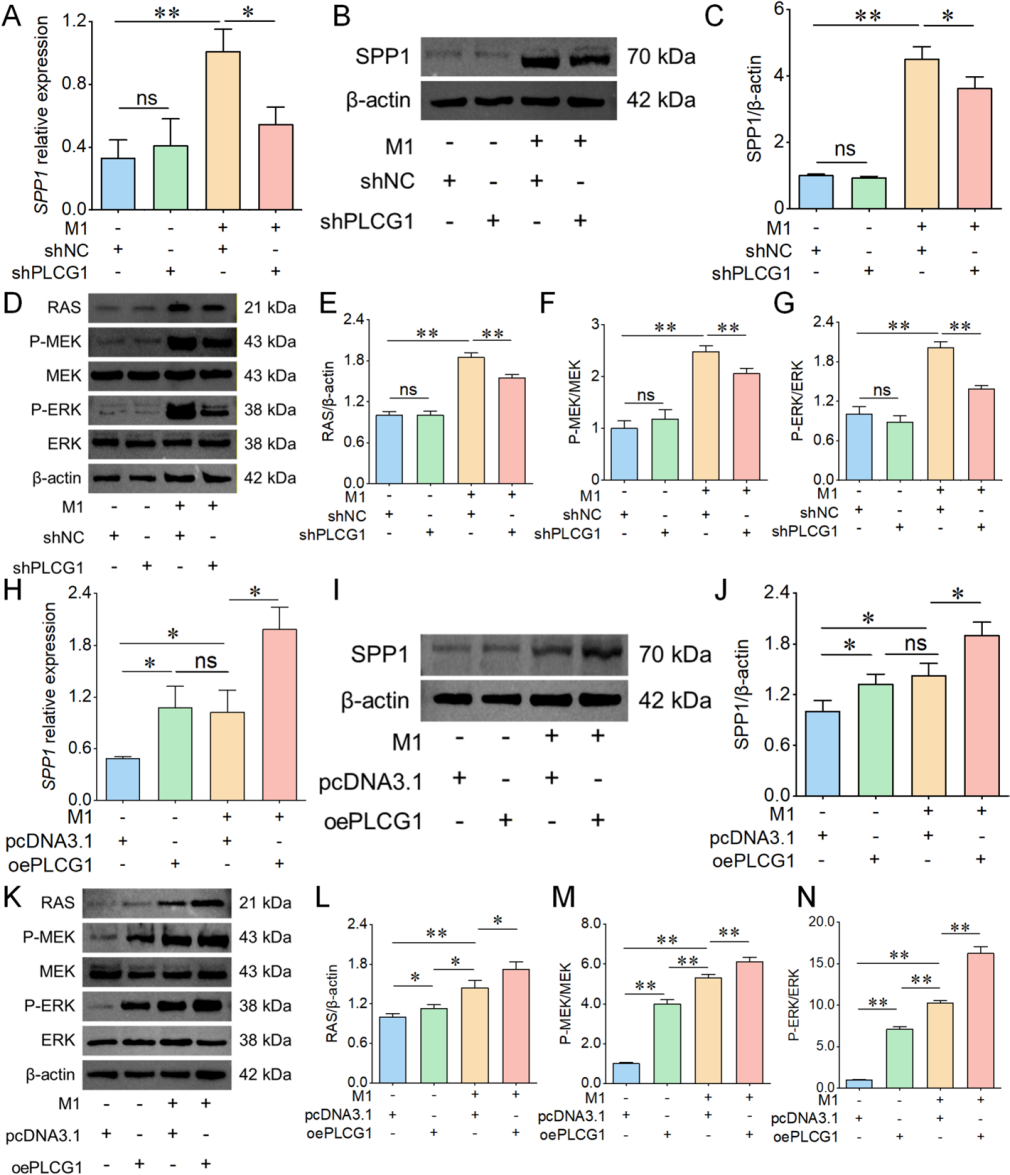
PLCG1 induces SPP1 production in M1 macrophages by activating the RAS/ERK pathway. We induced M1 polarization in THP-1 cells with LPS and IFN-γ and silenced or overexpressed PLCG1 to assess its impact on the RAS/ERK pathway and SPP1. **a**–**g** Silencing PLCG1 significantly downregulates *Spp1* gene expression (**a**), reduces SPP1 protein levels (**b**, **c**), and decreases RAS (**d**, **e**), P-MEK/MEK (**d**, **f**), and P-ERK/ERK (**d**, **g**) expression in M1 macrophages. **h**–**n** Overexpression of PLCG1 significantly upregulates *SPP1* gene expression (**h**), increases SPP1 protein levels (**i**, **j**), and increases RAS (**k**, **l**), P-MEK/MEK (**k**, **m**), and P-ERK/ERK (**k**, **n**) expression in M1 macrophages. *n* = 3 per group

PLCG1 is known to activate RAS [31]. Therefore, we hypothesized that PLCG1 activation in M1 macrophages could trigger the RAS/ERK signaling pathway, thereby inducing SPP1 expression. To investigate this, we silenced PLCG1 in THP-1 cells polarized into M1 macrophages and examined its effect on SPP1 expression. The results showed that PLCG1 silencing significantly reduced both the gene and protein expression of SPP1 (Fig. 7a–c). Similarly, the levels of RAS, P-MEK/MEK, and P-ERK/ERK were also significantly decreased (Fig. 7d–g). In contrast, after overexpressing PLCG1, both the gene and protein expression of SPP1 were significantly increased **(**Fig. 7h–j), along with elevated levels of RAS, P-MEK/MEK, and P-ERK/ERK (Fig. 7k–n). These findings confirm that PLCG1 regulates the RAS/ERK pathway to promote SPP1 production.

### ISOP targets PLCG1 to inhibit the RAS/ERK pathway, reducing SPP1 production and suppressing M1 macrophage migration and polarization

**Fig. 8:**
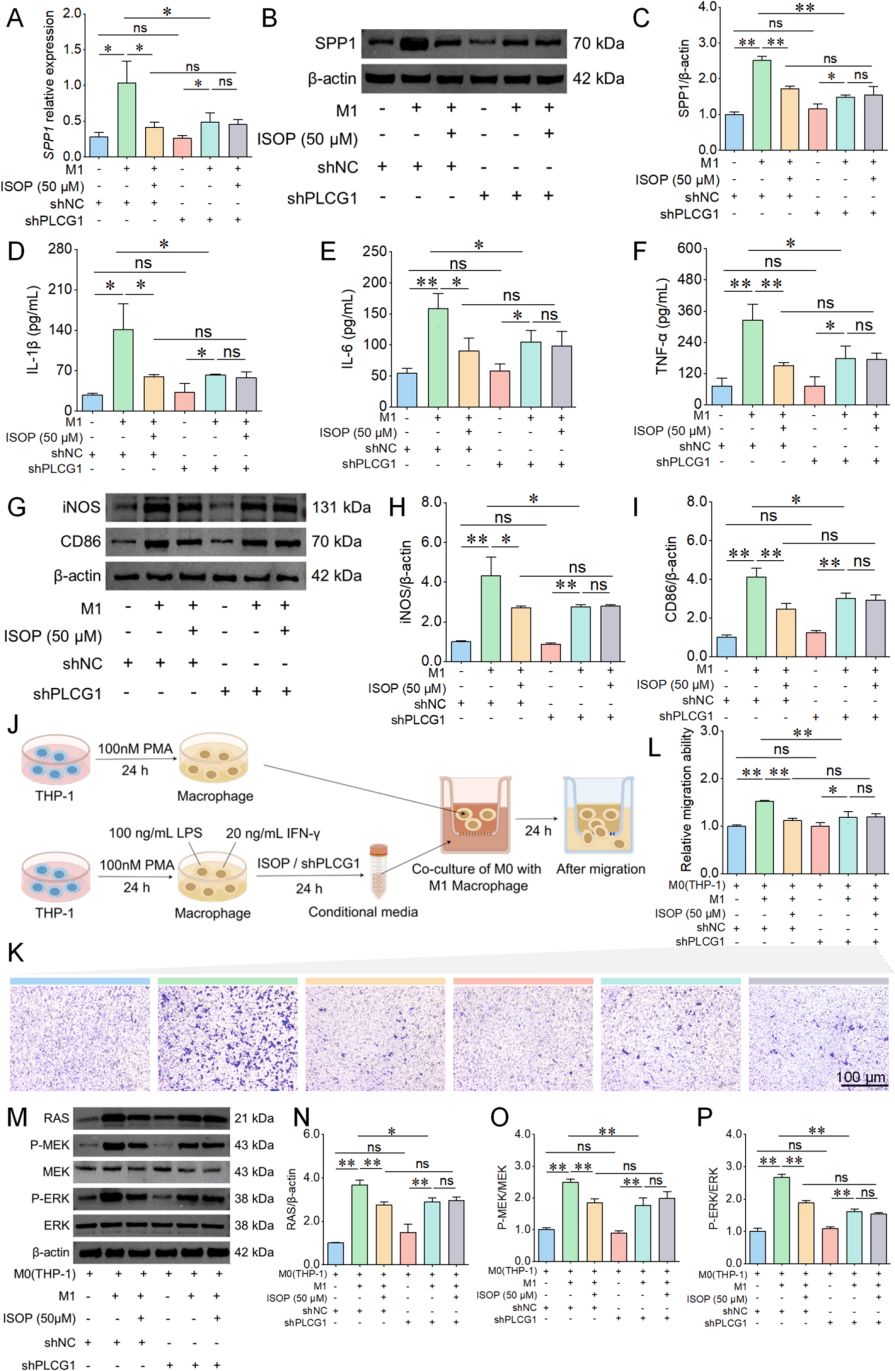
ISOP targets PLCG1 to inhibit RAS/ERK activation and subsequent SPP1 production. On the basis of in vitro PLCG1 silencing experiments, we further intervened with ISOP (50 μM) to explore the mechanism of ISOP inhibition in M1 migration and activation. **a–i** Both ISOP and shPLCG1 inhibits *SPP1* gene (**a**) and protein (**b**, **c**) expression in M1 macrophages, reduces IL-1β (**d**), IL-6 (**e**), and TNF-α (**f**) levels in supernatants, and downregulates iNOS (**g**, **h**) and CD86 (**g**, **i**) expression. No significant differences were observed between a combined ISOP and shPLCG1 intervention and a shPLCG1 intervention alone. **j** Schematic diagram of the Transwell experiment. **k–p** Transwell results showing both ISOP and shPLCG1 inhibits M1 macrophage migration. No significant differences were observed between a combined ISOP and shPLCG1 intervention and a shPLCG1 intervention alone (**k, l**). Both ISOP and shPLCG1 downregulates RAS (**m, n**), P-MEK/MEK (**m, o**), and P-ERK/ERK (**m, p**) expression. No significant differences were observed between a combined ISOP and shPLCG1 intervention and a shPLCG1 intervention alone. *n* = 3 per group

To confirm the mechanism by which ISOP inhibits the RAS/ERK pathway through targeting PLCG1, thereby reducing SPP1 production and suppressing M1 macrophage migration and polarization, we silenced PLCG1 in M1 macrophages and treated them with ISOP. Our results demonstrated that both ISOP and shPLCG1 suppressed the gene and protein expression of SPP1 in macrophages (Fig. 8a–c), reduced the levels of IL-1β, IL-6, and TNF-α in the supernatant (Fig. 8d–f), and downregulated the expression of iNOS and CD86 (Fig. 8g–i). Notably, the combined intervention with ISOP and shPLCG1 did not show a significant difference compared with shPLCG1 or ISOP alone in modulating these indicators. Additionally, Transwell assays revealed that both ISOP and shPLCG1 decreased the number of migrating macrophages, and the inhibitory effect of the combined ISOP and shPLCG1 treatment on cell migration was comparable to that of shPLCG1 or ISOP alone (Fig. 8j–l). Furthermore, ISOP and shPLCG1 significantly reduced the expression of RAS/ERK-related proteins, including RAS, P-MEK/MEK, and P-ERK/ERK. The combined ISOP and shPLCG1 intervention did not differ significantly from shPLCG1 or ISOP alone in suppressing these proteins (Fig. 8m–p).

### ISOP targets PLCG1 to inhibit M1 macrophage polarization and prevent RA-FLS proliferation and activation

**Fig. 9:**
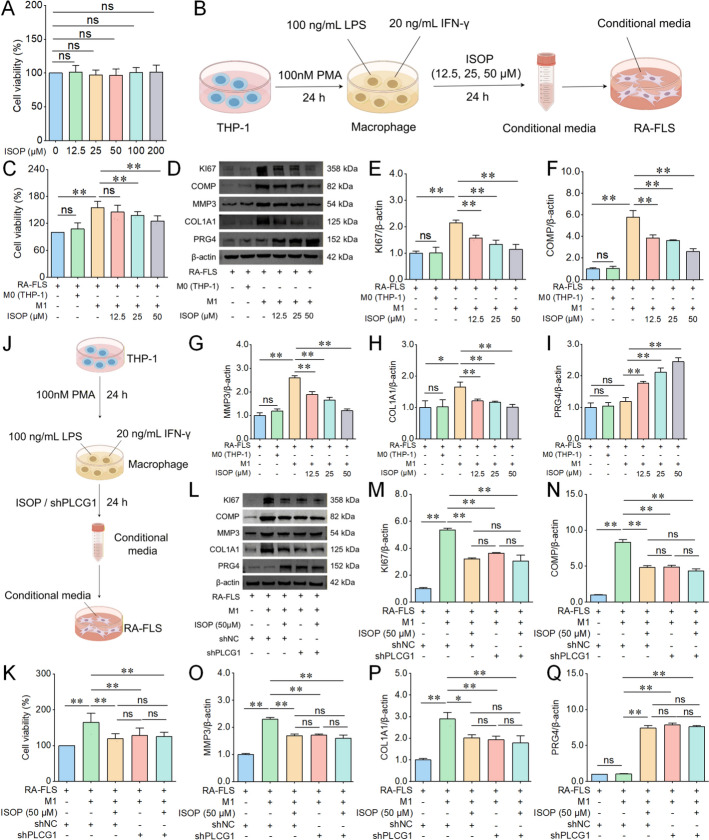
ISOP targets PLCG1 to inhibit M1 macrophage polarization, thereby blocking proliferation and activation of RA-FLS. We cultured MH7A cells in vitro and treated them with different concentrations of ISOP for 24 h to screen for noncytotoxic doses of RA-FLS. We also induced M1 polarization in THP-1 cells using PMA combined with LPS and IFN-γ, and applied 12.5, 25, and 50 μM ISOP or shPLCG1 interventions. Supernatants from each group of cells were collected for culturing of RA-FLS. **a** MTT results showing the cell viability of RA-FLS was not affected by ISOP intervention. **b** Schematic diagram of the experiment involving ISOP intervention in the coculture system of M1 macrophages and RA-FLS. **c** MTT results showing ISOP intervention dose-dependently reduces cell viability of RA-FLS in co-culture system. **d**–**i** ISOP intervention downregulates expression of KI67 (**d**, **e**), COMP (**d**, **f**), MMP3 (**d**, **g**), and COL1A1 (**d**, **h**) proteins to varying degrees, and upregulates expression of the PRG4 (**d**, **i**) protein in co-culture system. **j** Schematic diagram of the experiment involving ISOP or shPLCG1 interventions in the coculture system of M1 macrophages and MH7A cells. **k** MTT results showing both ISOP and shPLCG1 interventions reduces cell viability of RA-FLS in co-culture system. No significant differences were observed between a combined intervention of ISOP and shPLCG1 and a shPLCG1 intervention alone.** l**–**q** Both ISOP and shPLCG1 interventions downregulates expression of KI67 (**l**, **m**), COMP (**l**, **n**), MMP3 (**l**, **o**), and COL1A1 (**l**, **p**) proteins and upregulates expression of PRG4 (**l**, **q**) protein. No significant differences were observed between a combined intervention of ISOP and shPLCG1 and a shPLCG1 intervention alone. *n* = 3 per group

Our findings suggest that ISOP exerts its anti-inflammatory effects by inhibiting SPP1 production in M1 macrophages. Additionally, the abnormal proliferation and activation of FLS into RA-FLS are key pathological features of RA synovial inflammation. Consistent with scRNA-seq results, ISOP intervention reduced the relative percentage of RA-FLS (Fig. 2g). To further investigate ISOP’s effect on RA-FLS proliferation and activation, we conducted additional experiments. Interestingly, direct ISOP treatment did not influence RA-FLS proliferation (Fig. 9a). Previous studies have shown that SPP1 induces RA-FLS proliferation and activation, thereby contributing to RA progression [32]. On this basis, we hypothesized that ISOP indirectly affects RA-FLS by targeting macrophages. To test this hypothesis, we cultured RA-FLS with the supernatant from THP-1 cells treated with ISOP (Fig. 9b). Culturing RA-FLS with the supernatant of THP-1 cells polarized into M1 macrophages enhanced RA-FLS viability (Fig. 9c) and increased the expression of proliferation- and activation-related proteins KI67, COMP, MMP3, and COL1A1 (Fig. 9d–i). However, when RA-FLS were cultured with the supernatant of THP-1 cells treated with ISOP, their viability decreased (Fig. 9c). This treatment also downregulated KI67, COMP, MMP3, and COL1A1 expression while upregulating PRG4 expression (Fig. 9d–i).

To further verify the mechanism by which ISOP targets PLCG1 in macrophages to inhibit RA-FLS proliferation and activation, we silenced PLCG1 in macrophages (Fig. 9j). The results showed that the supernatant of THP-1 cells treated with ISOP or shPLCG1 suppressed RA-FLS proliferation (Fig. 9k) and reduced the expression of proliferation- and activation-related proteins KI67, COMP, MMP3, and COL1A1, while increasing PRG4 expression (Fig. 9l–q). Importantly, no significant differences were observed between the shPLCG1 and ISOP + shPLCG1 intervention groups.

## Discussion

In this study, we assessed the therapeutic potential of ISOP for RA using the CIA rat model, a widely accepted model that mimics the clinical symptoms, pathological changes, and immune responses observed in patients with RA [33]. Our results revealed that rats in the model group developed joint swelling, abnormal fibroblast proliferation in the synovial membrane, inflammatory cell infiltration, and other pathological features characteristic of RA synovial inflammation. Typically, an arthritis index (sum of scores for all four limbs) of ≥ 4 was considered indicative of successful model induction [34]. Our results showed that arthritis indices exceeded 4 on day 21 following CII/IFA immunization, confirming successful model establishment. ISOP treatment produced significant therapeutic effects in RA, improving pathological damage such as synovial hyperplasia, joint space narrowing, and inflammatory infiltration. MTX was chosen as the positive control, given its proven efficacy in the clinical management of RA [35]. Notably, high-dose ISOP demonstrated a therapeutic effect comparable to that of MTX, alleviating joint pathological damage in CIA rats, thus further supporting ISOP’s anti-RA potential.

Next, we performed scRNA-seq to examine the transcriptional landscape of synovial tissues in the Control, CIA, and ISOPH groups. We classified cells into five categories: myeloid cells, lymphoid cells (immune cells), and fibroblasts, endothelial cells, and neurocytes (nonimmune cells). Our analysis revealed a significant increase in the relative proportion of immune cells in the CIA group, suggesting that immune cell infiltration predominates in the synovial tissue of CIA rats. ISOP intervention reduced the percentage of immune cells in synovium, leading us to hypothesize that immune cells are the primary targets of ISOP. The synovium is primarily composed of macrophages (type A synovial cells) and FLS (type B synovial cells) [36]. Our results also revealed that macrophages and synovial fibroblasts represented the majority of immune and nonimmune cells, respectively. Since ISOP may target immune cells, we focused on macrophages, particularly *Spp1*^+^ macrophages (C03), which exhibited the most significant changes following ISOP treatment. Therefore, we hypothesized that ISOP targets *Spp1*^+^ macrophages to exert its anti-RA effects.

SPP1, also known as osteopontin (OPN), is a phosphorylated, sialic acid-rich, non-collagenous bone matrix protein and a member of the SIBLING family. It is widely expressed in various cell types, including macrophages [37]. SPP1 plays a critical role in immune responses by binding to CD44 receptors, mediating cell-to-cell and cell-to-matrix interactions, and promoting the progression of numerous diseases, such as lung cancer [38], cardiovascular diseases [37], and metabolic disorders such as nonalcoholic steatohepatitis [39]. SPP1 is particularly overexpressed in RA [40]. Previous studies have shown that pro-inflammatory cytokines secreted by M1 macrophages, such as IL-1β, IL-6, and TNF-α, stimulate SPP1 secretion, which further drives macrophage migration and activation toward inflammatory sites, contributing to cytokine storm formation [41]. In our study, we observed a significant increase in *Spp1*^+^ macrophages in the synovial membranes of CIA rats. Furthermore, after LPS and IFN-γ induced M1 polarization in THP-1 cells, SPP1 expression was markedly upregulated. This upregulation was accompanied by increased levels of M1-related markers, including IL-1β, IL-6, and TNF-α, as well as higher expression of iNOS, CD86, CD44, and chemokines, and an increased number of M1 macrophage migrations. These findings highlight the crucial role of SPP1 in regulating M1 macrophages and its significant involvement in RA progression. Importantly, ISOP intervention reversed these effects, suggesting its inhibitory impact on M1 macrophage migration, activation, and SPP1 secretion.

In subsequent studies, we observed that ISOP intervention downregulated the expression of several genes and proteins within the RAS/ERK pathway in macrophages. ERK, a key member of the MAPK family, plays a critical role in regulating SPP1 production [29]. Research has shown that ERK is overexpressed in RA [42], and inhibiting its phosphorylation can effectively reduce pro-inflammatory cytokine levels and alleviate synovial inflammation in RA [43]. Further investigation revealed that ISOP also downregulated the expression of RAS-related genes, which are essential upstream regulators of ERK. Extensive studies have demonstrated that RAS acts as a major upstream activator of ERK [43], suggesting that the activation of the RAS/ERK pathway in M1 macrophages promotes SPP1 production. RAS is often referred to as the “signaling switch” in RA progression; its overexpression enhances the secretion of pro-inflammatory cytokines, which exacerbates inflammation and fibrosis [44]. Additionally, RAS can activate MEK, which in turn promotes ERK phosphorylation, driving subsequent immune responses [29, 45–48]. MEK functions as a critical intermediary between RAS and ERK and has been identified as a key target in RA pathogenesis [49]. By receiving upstream RAS phosphorylation signals, MEK mediates downstream ERK phosphorylation, regulating immune cell migration and activation, and further promoting inflammatory responses [50]. Targeting the RAS/MEK/ERK pathway has proven to be an effective strategy for alleviating RA [43].

Further investigation revealed that PLCG1 is a direct target of ISOP in inhibiting the activation of the RAS/ERK pathway. PLCG1 is a key upstream driver in the pathogenesis of immune dysregulation diseases, particularly RA [51], and its phosphorylation level is directly linked to abnormal articular cartilage structure [52]. Studies have shown that excessive activation of PLCG1 catalyzes the formation of inositol 1,4,5-trisphosphate and diacylglycerol from phosphatidylinositol 4,5-bisphosphate. This process activates RAS guanyl-releasing protein 1 and protein kinase C, which in turn promotes the overactivation of RAS and aberrantly activates the RAS/ERK pathway, exacerbating inflammatory cytokine storms and worsening synovial inflammation in the joints [31, 53]. Consequently, PLCG1 is considered a potential therapeutic target for arthritis [54]. Existing research has identified PLCG1 as a key marker for predicting RA progression [55]. Moreover, studies have demonstrated that inhibiting PLCG1 exerts chondroprotective effects by modulating ERK-related pathways [30], and preventing the development of early osteoclasts [28], making it a promising strategy for RA treatment. PLCG1 (PLCγ1) contains eight domains, four of which are unique to the PLC family. These unique domains are referred to as “γ1 specific array” domains [56]. The “γ1 specific array” domains comprise a “split” PH domain flanking two tandem SH2 (nSH2 and cSH2) domains and an SH3 domain. According to our docking results, hydrogen-bond interactions were observed between Met750, Lys749 and ISOP. Interestingly, Lys749 and Met750 are located in the cSH2 domain. Previous studies have shown that SH2 domains mediate the phosphorylation of PLCG1[56]. In line with this, our results demonstrated that ISOP suppressed PLCG1 phosphorylation. Moreover, the cSH2 domain serves as a central regulatory region of PLCG1 [56]. Therefore, the binding sites obtained from the docking results are likely to be the activation site of PLCG1. ISOP may occupy the active site cavity of PLCG1, thereby inhibiting its activation. Future study will be conducted to validate the binding sites between ISOP and PLCG1 using point mutation experiments. Besides, silencing PLCG1 significantly decreased the expression of RAS/ERK-related proteins, downregulated M1 macrophage markers and SPP1 expression, and reduced the migration of M1 macrophages. Notably, ISOP exerted effects similar to PLCG1 silencing, suggesting that ISOP inhibits the RAS/ERK pathway by targeting PLCG1. This results in reduced SPP1 production and diminished M1 macrophage migration and activation.

Our findings confirmed that ISOP effectively inhibits RA-FLS proliferation. FLS are key producers of essential components in synovial fluid and stroma, playing a vital role in maintaining joint homeostasis [57]. However, under pathological conditions, interactions between macrophages and FLS drive cytokine storm formation, promoting the transformation of FLS into RA-FLS and exacerbating joint inflammation and synovial damage [58, 59]. ISOP intervention significantly suppressed the expression of RA-FLS proliferation-related proteins, including KI67, COMP, MMP-3, and COL1A1, while upregulating the synovial protection-related protein PRG4. These results highlight the antiproliferative effects of ISOP on RA-FLS. Notably, direct ISOP treatment did not affect RA-FLS proliferation, indicating that ISOP exerts its effects indirectly. Given the complexity of RA development, it involves intricate cellular interactions and networks, and the well-documented connection between macrophages and RA-FLS [60]. Our results have identified macrophages as key target cells of ISOP. Therefore, we hypothesized that macrophages mediate the anti-RA-FLS effects of ISOP. To explore this hypothesis, we established an in vitro co-culture system of M1 macrophages and RA-FLS. ISOP treatment reversed M1 polarization and concurrently inhibited RA-FLS proliferation. These effects were comparable to those observed with PLCG1 silencing, suggesting that ISOP targets PLCG1-mediated SPP1 production from M1 macrophages to suppress RA-FLS proliferation.

Interestingly, ISOP shows a chemical core structure like imperatorin. Previous studies have revealed the anti-inflammatory effects of imperatorin on primary human osteoarthritis chondrocytes [61]. Future studies will focus on the therapeutic effect of ISOP on osteoarthritis. In addition, the effect of ISOP on chondrocytes warrants further investigation. Meanwhile, the anti-RA effect of imperatorin has also been reported [62], and comparison study could be carried out to compare the anti-RA efficacy and safety between imperatorin and ISOP. Besides, imperatorin has been demonstrated as a PDE4B inhibitor [63]. Whether the anti-inflammatory effect of ISOP is mediated by PDE4B activation remains to be determined. ISOP has also structural similarity to psoralen. As psoralen undergoes photochemical reaction through interacting with DNA under exposure to ultraviolet radiation (UV) [64], future studies will assess whether ISOP undergoes photochemical reactions upon UV exposure.

This study has several limitations. First, we used Sprague Dawley (SD) rats to evaluate the anti-RA effects of ISOP. Although SD rats have been used in CIA model establishment [34, 65], Dark Agouti (DA), Louvain (LOU), BioBreeding (BB), and Lewis (LEW) rats are more commonly employed for CIA model induction [66–69]. Future studies will employ CIA-susceptible rat strains (DA, LOU, BB, and LEW) to evaluate the therapeutic potential of ISOP on RA. Second, although TPP, molecular docking, CETSA, and DARTS indicated the possible binding between PLCG1 and ISOP, the direct binding between ISOP and PLCG1 remains unclear. Future studies will employ physicochemical methods, such as surface plasmon resonance (SPR) and bio-layer interferometry (BLI), to directly assess the binding affinity between ISOP and PLCG1. Moreover, PLCG1 is known to be activated through phosphorylation [70, 71]. Consistently, our results showed that ISOP inhibited PLCG1 phosphorylation. These data suggest that ISOP may inhibit the enzymatic activity of ISOP. The inhibitory effect of ISOP on PLCG1 enzymatic activity will be validated using biochemical methods.

## Conclusions

**Fig. 10:**
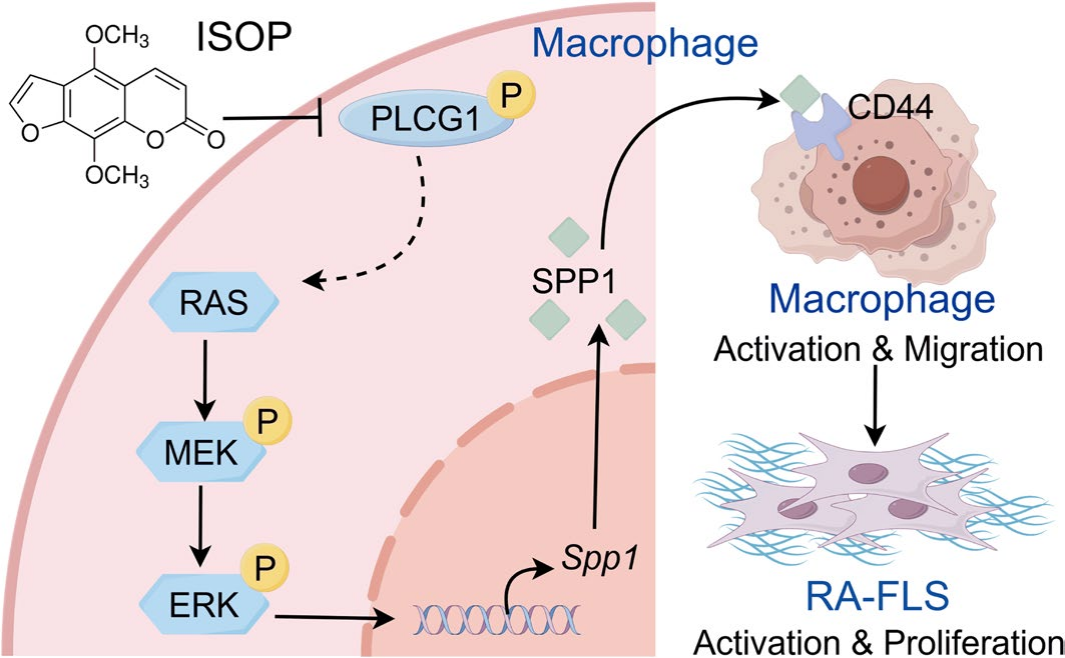
Schematic diagram of the mechanisms in this study. ISOP inhibits the activation of the RAS/ERK pathway by targeting PLCG1 and reduces SPP1 production in M1 macrophages, thereby blocking the proliferation and activation of RA-FLS, and alleviates synovial inflammation

ISOP shows significant therapeutic potential for RA. Mechanistic studies, including scRNA-seq and in vitro validation, demonstrate that ISOP inhibits the activation of the RAS/ERK pathway by targeting PLCG1. This action reduces SPP1 secretion by M1 macrophages and inhibits the proliferation and activation of RA-FLS (Fig. 10). Our study is the first to elucidate the mechanism by which PLCG1 regulates SPP1 production in M1 macrophages and identifies key targets of ISOP in RA treatment from multiple perspectives. These findings provide a scientific basis for the clinical application of ISOP and the advancement of RA diagnostic and therapeutic strategies.

## Supplementary Information

Additional file 1.

Additional file 2.

## Data Availability

The data that support the findings of this study are available from the corresponding author upon reasonable request.

## Abbreviations

BB: BioBreeding
BLI: Bio-layer interferometry
CETSA: Cellular thermal shift assays
CIA: Collagen-induced arthritis
CII: Bovine type II collagen
DA: Dark Agouti
DARTS: Drug affinity responsive target stability
DEGs: Differentially expressed genes
FLS: Fibroblast-like synoviocytes
GO: Gene Ontology
IFA: Incomplete Freund’s adjuvant
ISOP: Isopimpinellin
KEGG: Kyoto Encyclopedia of Genes and Genomes
LEW: Lewis
LOU: Louvain
MTX: Methotrexate
OPN: Osteopontin
PLCG1: Phospholipase Cgamma1
RA: Rheumatoid arthritis
scRNA-seq: Single-cell RNA sequencing
SD: Sprague Dawley
SPP1nAb: SPP1 neutralizing antibody
SPR: Surface plasmon resonance
TPP: Thermal proteome profiling
UV: Ultraviolet radiation
