# Supplementary material for: Integrated analysis of single-cell RNA sequencing, transcriptomics, and thermal proteome profiling identifies PLCG1 as the therapeutic target of isopimpinellin in treating rheumatoid arthritis
Source: Cell Mol Biol Lett. 2026 Apr 4;31:89. doi: 10.1186/s11658-026-00918-8 (PMC13281331; doi:10.1186/s11658-026-00918-8)
Supplement: Supplementary file 1 — Additional file 1. [file 11658_2026_918_MOESM1_ESM.docx]

***Supplementary Materials***

**1 Reagents**

Isopimpinellin (ISOP, B21533) was purchased from Shanghai Yuanye Bio-Technology Co., Ltd. (Shanghai, China). Incomplete Freund’s adjuvant (IFA, Catalog#7002) and bovine Type II collagen (CII, 234184-M) were bought from Sigma (Shanghai, China). Methotrexate (MTX, S18026), PMA (R32414) and lipopolysaccharide (LPS, S11060) were purchased from YuanYe Bio-Technology (Shanghai, China). HE staining assay kit ([D006-1-1](http://www.njjcbio.com/products.asp?id=476)) was purchased from Nanjing Jiancheng Biological Engineering Institute (Nanjing, China). Interferon-γ (IFN-γ, 400-20) was purchased from PeproTech China (Suzhou, China). Brefeldin A (BFA, B8581), Thiazolyl blue tetrazolium bromide (MTT, M8180) were bought from Beijing Solarbio Science & Technology Co.,Ltd. (Beijing, China). ELISA kits of human TNF-α (ml077385), IL-1β (ml058059), IL-6 (ml059930) were bought from Enzyme-linked Biotechnology (Shanghai, China). ELISA kits of rat TNF-α (ml002953), IL-1β (ml107050), IL-6 (ml106838) were bought from Shanghai Enzyme-linked Biotechnology Co., Ltd. (Shanghai, China). Pronase E (HY-114158) was purchased from Shanghai Medchemexpress Bio-Technology Co., Ltd. (Shanghai, China). iNOS (ab178945), CD11b (ab1211) were purchased from Abcam (Shanghai, China). SPP1 (22952-1-AP), RAS (81615-1-RR), P-ERK (28733-1-AP), ERK (11257-1-AP), KI67 (28074-1-AP), COMP (28369-1-AP), MMP3 (17873-1-AP), COL1A1 (67288-1-Ig) were purchased from Proteintech Technology (Wuhan, China). PLCG1 (bs-3533R Bioss) were purchased from bioss Bio-Technology Co., Ltd. (Beijing, China). P-MEK (#9154), MEK (#8727) were purchased from Cell Signaling Technology (Shanghai, China). PRG4(CAB15379) were purchased from Amyjet scientific Co., Ltd. (Wuhan, China).

**2 Arthritis Index Scoring Criteria**

0 points for no joint redness or swelling; 1 point for mild swelling or local redness in the toe joints; 2 points for swelling or redness extending from the toes to the dorsum of the foot; 3 points for redness and swelling of the ankle joint and below, with mild limitation of movement; and 4 points for severe redness and swelling with marked limitation of movement. A cumulative score for the four limbs was used as final results. The arthritis index score ≥ 4 was considered to be a successful model.

**3 Pathological Evaluation**

Soft tissue around the joint: 0 points: No abnormalities. 1 point: Mild inflammation. 2 points: Moderate inflammation. 3 points: Severe inflammation.

Synovial tissue: 0 points: No abnormalities. 1 point: Mild inflammation. 2 points: Moderate inflammation. 3 points: Severe inflammation.

Cartilage tissue: 0 points: No abnormalities. 1 point: Mild articular cartilage damage and intra-articular pannus formation. 2 points: Articular cartilage destruction, localized cartilage loss, fibrous tissue hyperplasia, and infiltration of neutrophils and lymphocytes. 3 points: Severe articular cartilage damage, extensive fibrous tissue hyperplasia, inflammation throughout the joint cavity, and substantial infiltration of neutrophils and lymphocytes.

**4 Supplemental Figures**


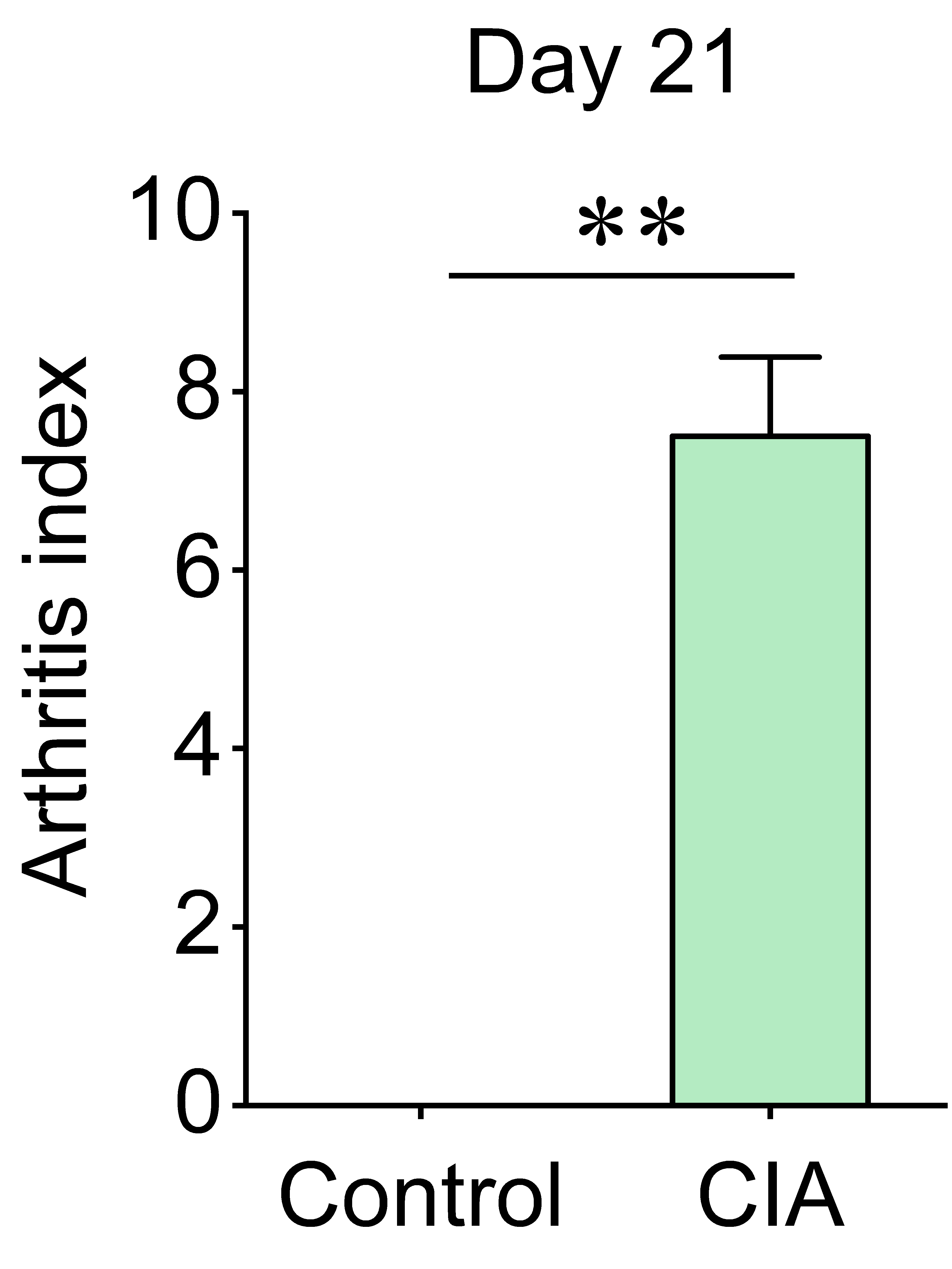


**Fig. S1** Arthritis index on day 21 after CII/IFA administration. *n* = 10 for Control group and *n* = 50 for CIA group. ***P* < 0.01.


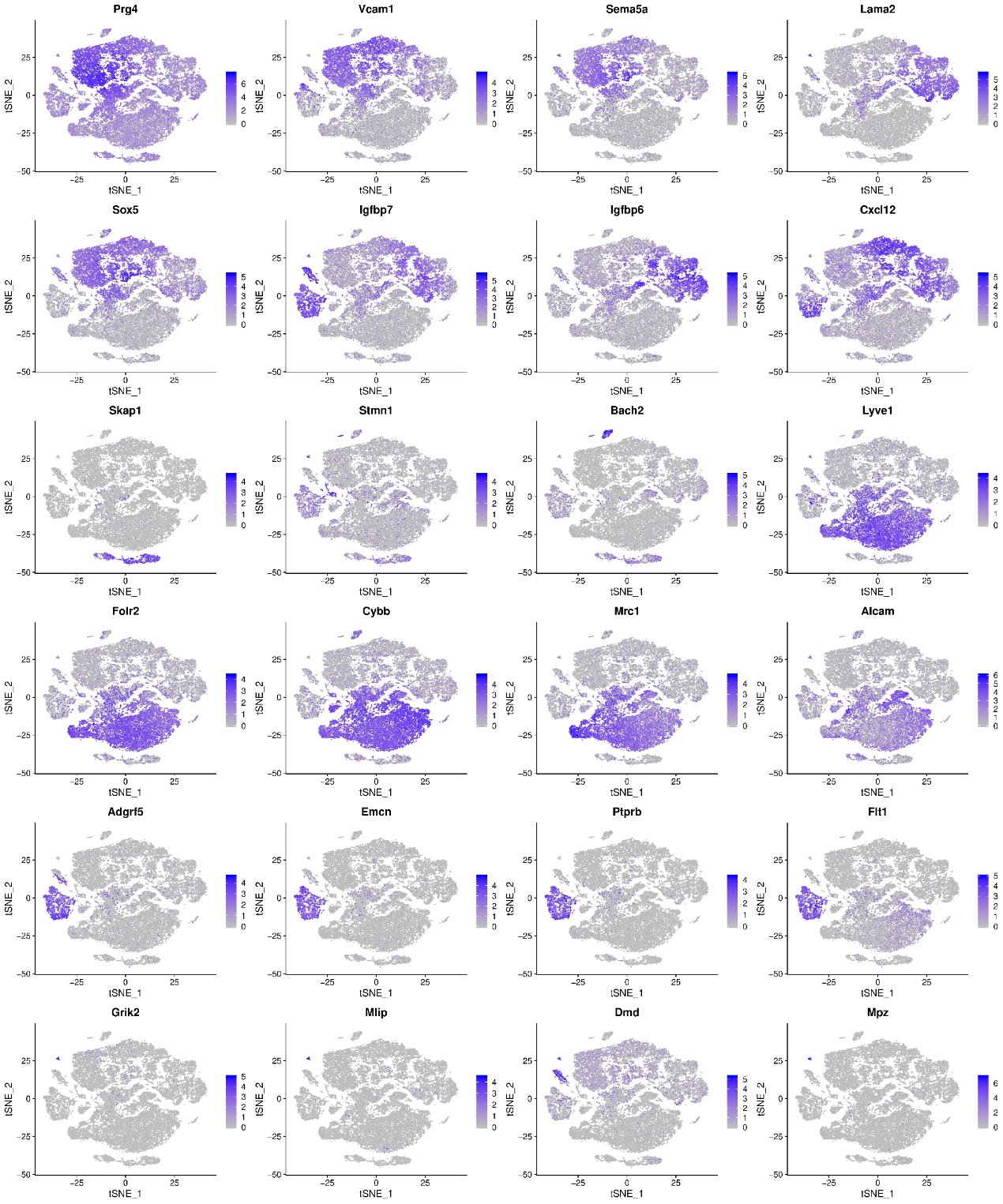


**Fig. S2** Marker Gene Distributions of Myeloid, Lymphoid, Fibroblast, Endothelial, and Nerve cells based on t-SNE.


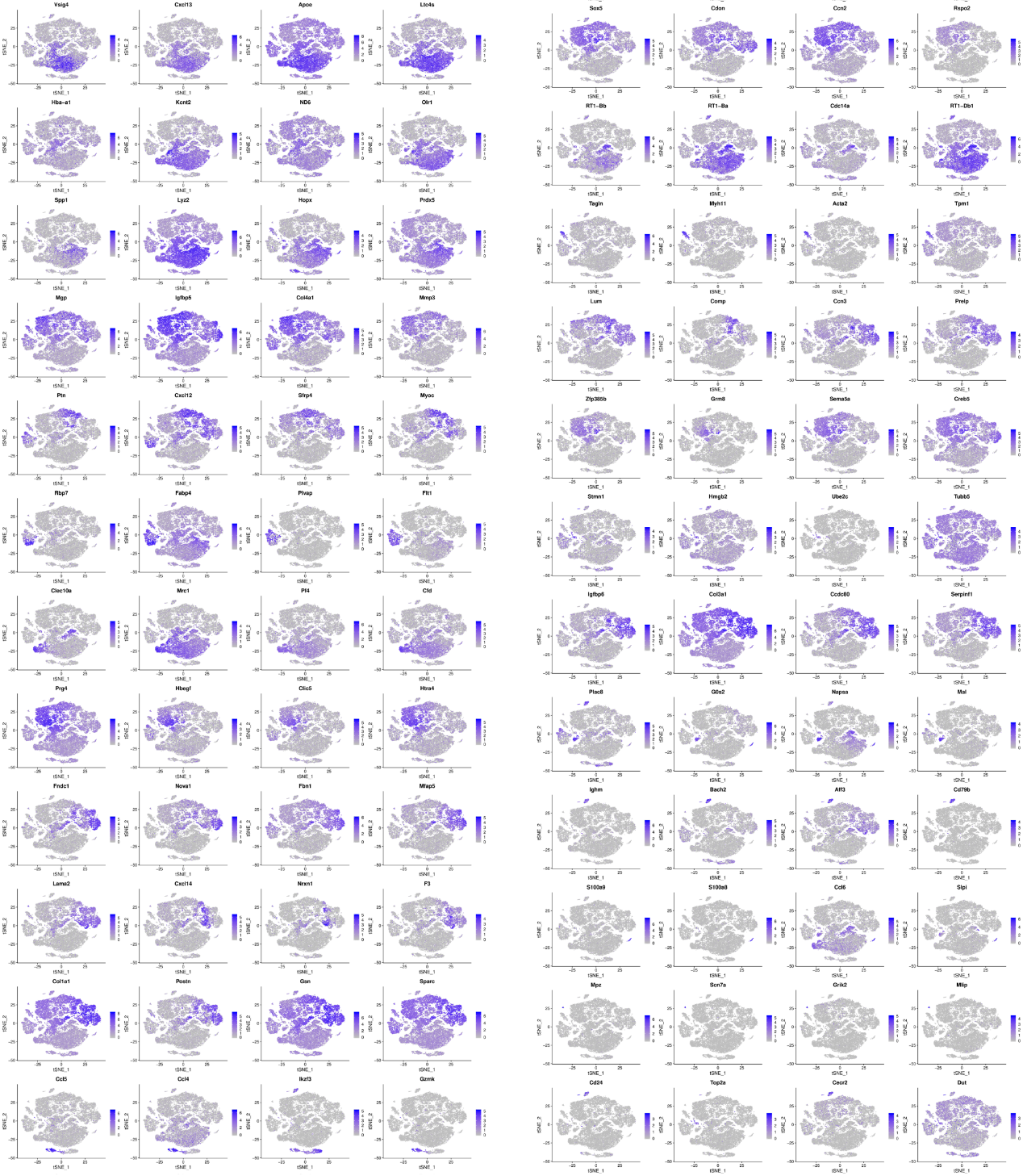


**Fig. S3** Marker Gene Distributions of the 24 Clusters based on t-SNE.


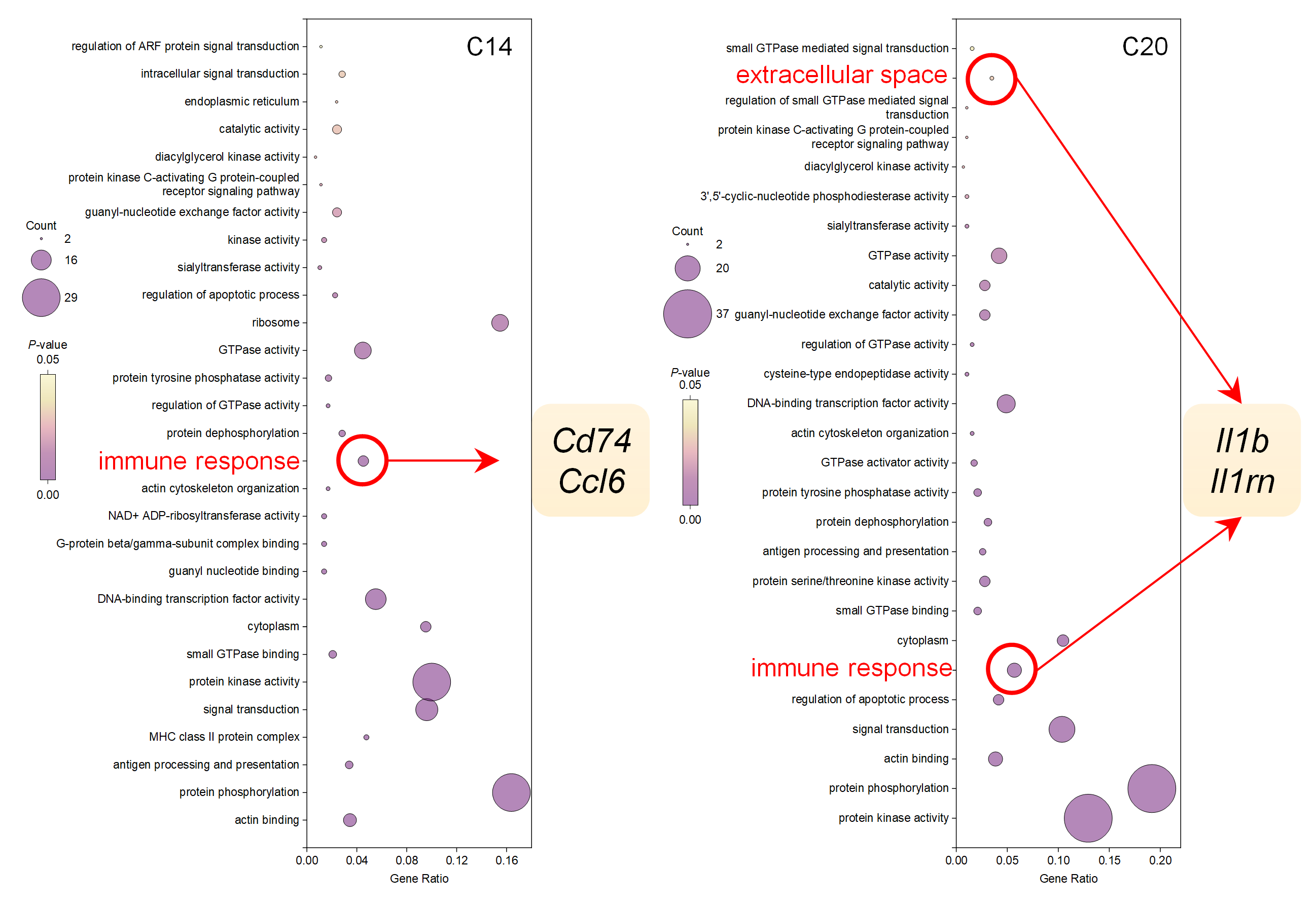


**Fig. S4** GO Analysis of Marker Genes for C14 and C20.
