# Supplementary material for: Integrated analysis of single-cell RNA sequencing, transcriptomics, and thermal proteome profiling identifies PLCG1 as the therapeutic target of isopimpinellin in treating rheumatoid arthritis
Source: Cell Mol Biol Lett. 2026 Apr 4;31:89. doi: 10.1186/s11658-026-00918-8 (PMC13281331; doi:10.1186/s11658-026-00918-8)

Figure 3G

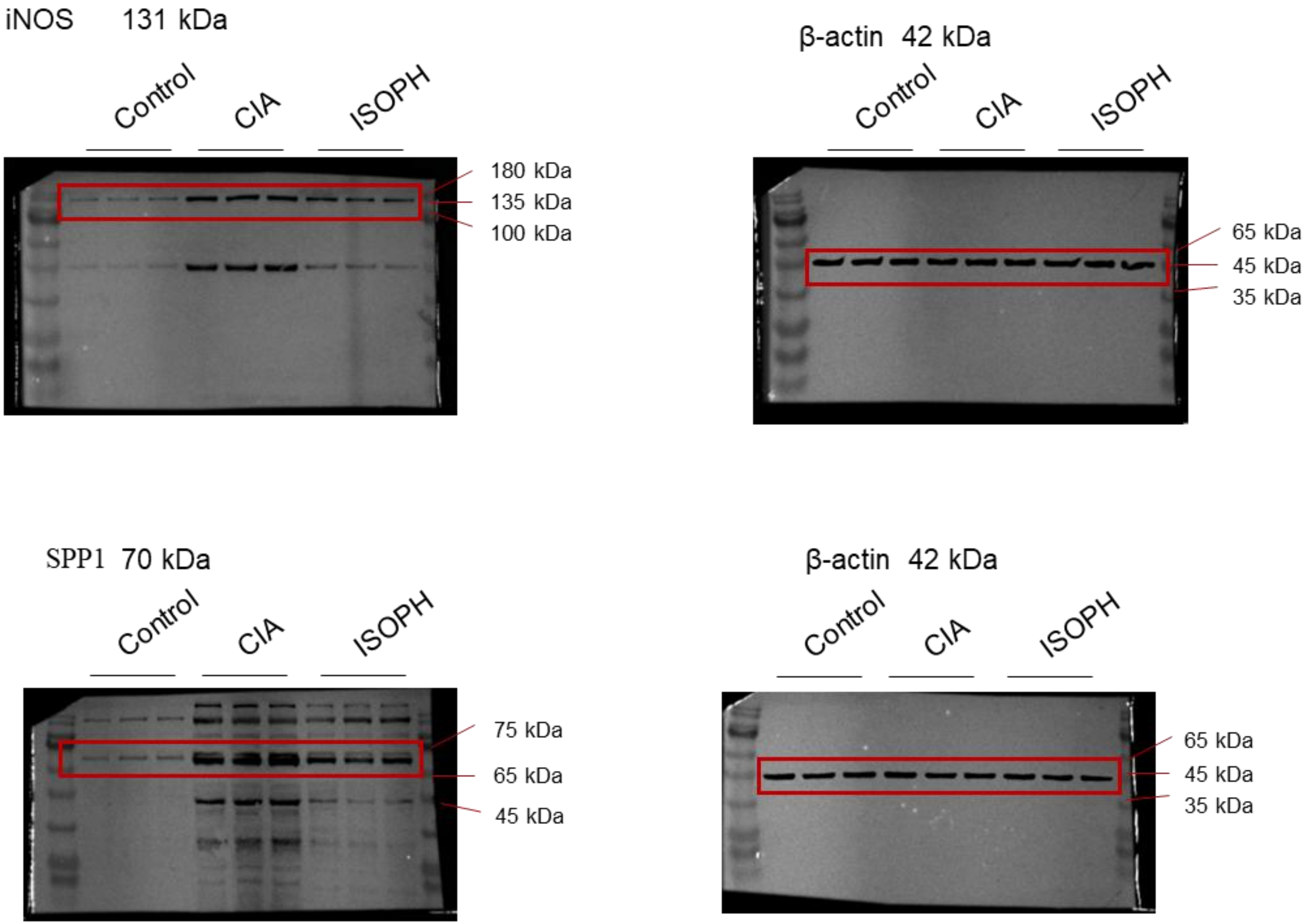

iNOS 131 kDa
Control
CIA
ISOPH
180 kDa
135 kDa
100 kDa
β-actin 42 kDa
Control
CIA
ISOPH
65 kDa
45 kDa
35 kDa
SPP1 70 kDa
Control
CIA
ISOPH
75 kDa
65 kDa
45 kDa
β-actin 42 kDa
Control
CIA
ISOPH
65 kDa
45 kDa
35 kDa

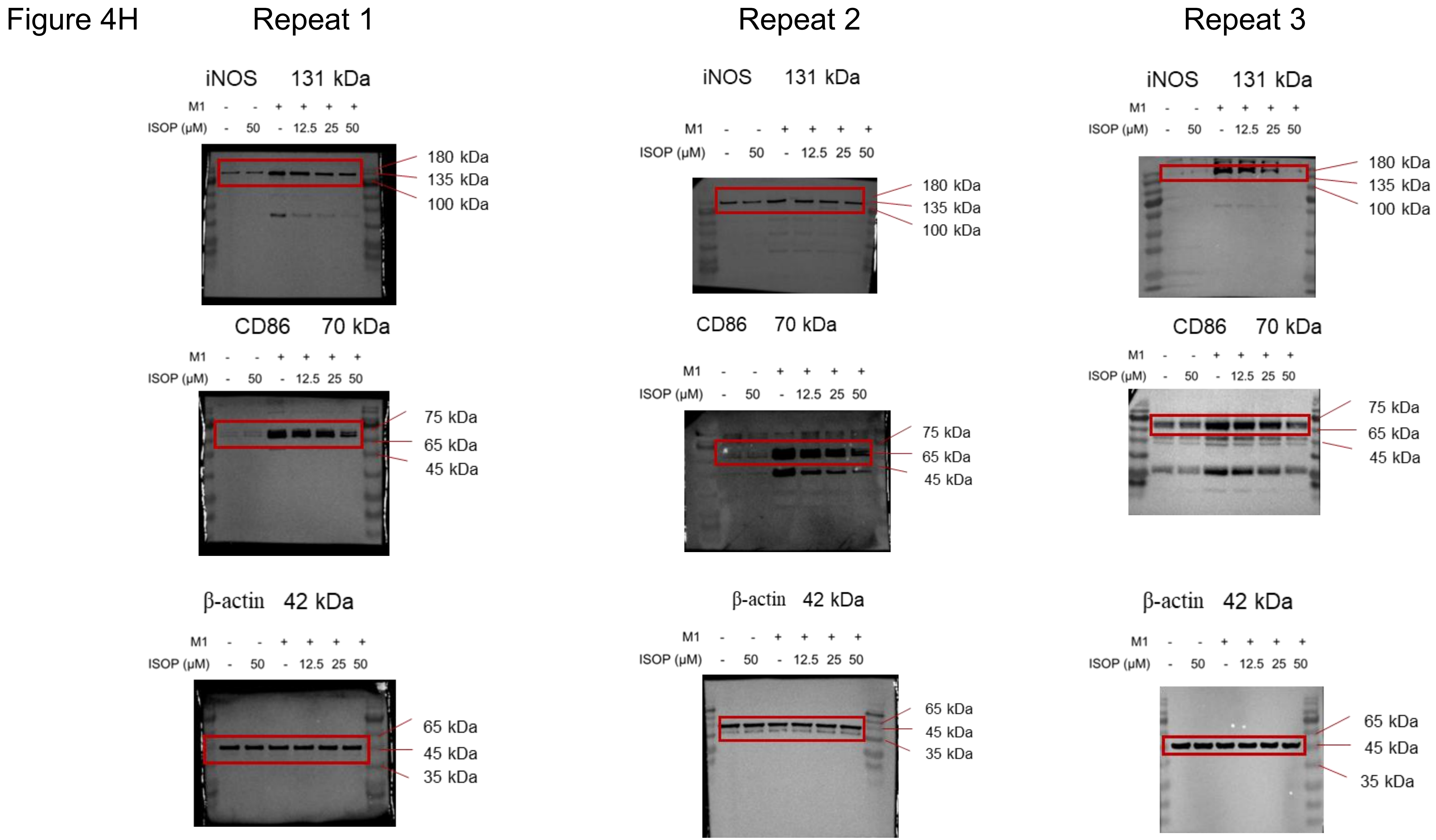
Figure 4H
Repeat 1
Repeat 2
Repeat 3
iNOS 131 kDa
M1 - - + + + +
ISOP (μM) - 50 - 12.5 25 50
180 kDa
135 kDa
100 kDa
CD86 70 kDa
75 kDa
65 kDa
45 kDa
β-actin 42 kDa
35 kDa

Figure 5C

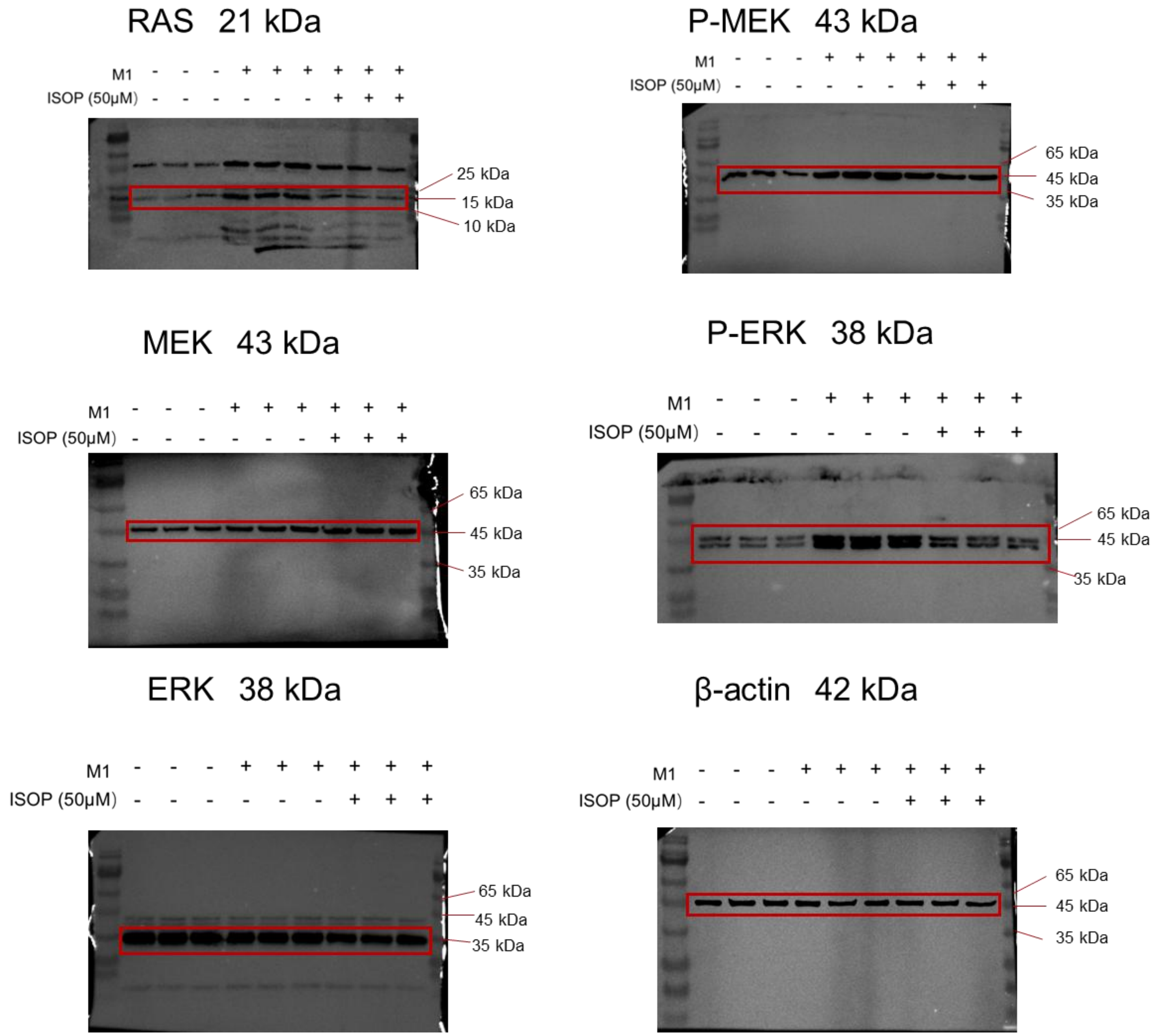
RAS 21 kDa
M1 - - - + + + + + +
ISOP (50μM) - - - - - - + + +
25 kDa
15 kDa
10 kDa
P-MEK 43 kDa
M1 - - - + + + + + +
ISOP (50μM) - - - - - - + + +
65 kDa
45 kDa
35 kDa
MEK 43 kDa
M1 - - - + + + + + +
ISOP (50μM) - - - - - - + + +
65 kDa
45 kDa
35 kDa
P-ERK 38 kDa
M1 - - - + + + + + +
ISOP (50μM) - - - - - - + + +
65 kDa
45 kDa
35 kDa
ERK 38 kDa
M1 - - - + + + + + +
ISOP (50μM) - - - - - - + + +
65 kDa
45 kDa
35 kDa
β-actin 42 kDa
M1 - - - + + + + + +
ISOP (50μM) - - - - - - + + +
65 kDa
45 kDa
35 kDa

Figure 6E

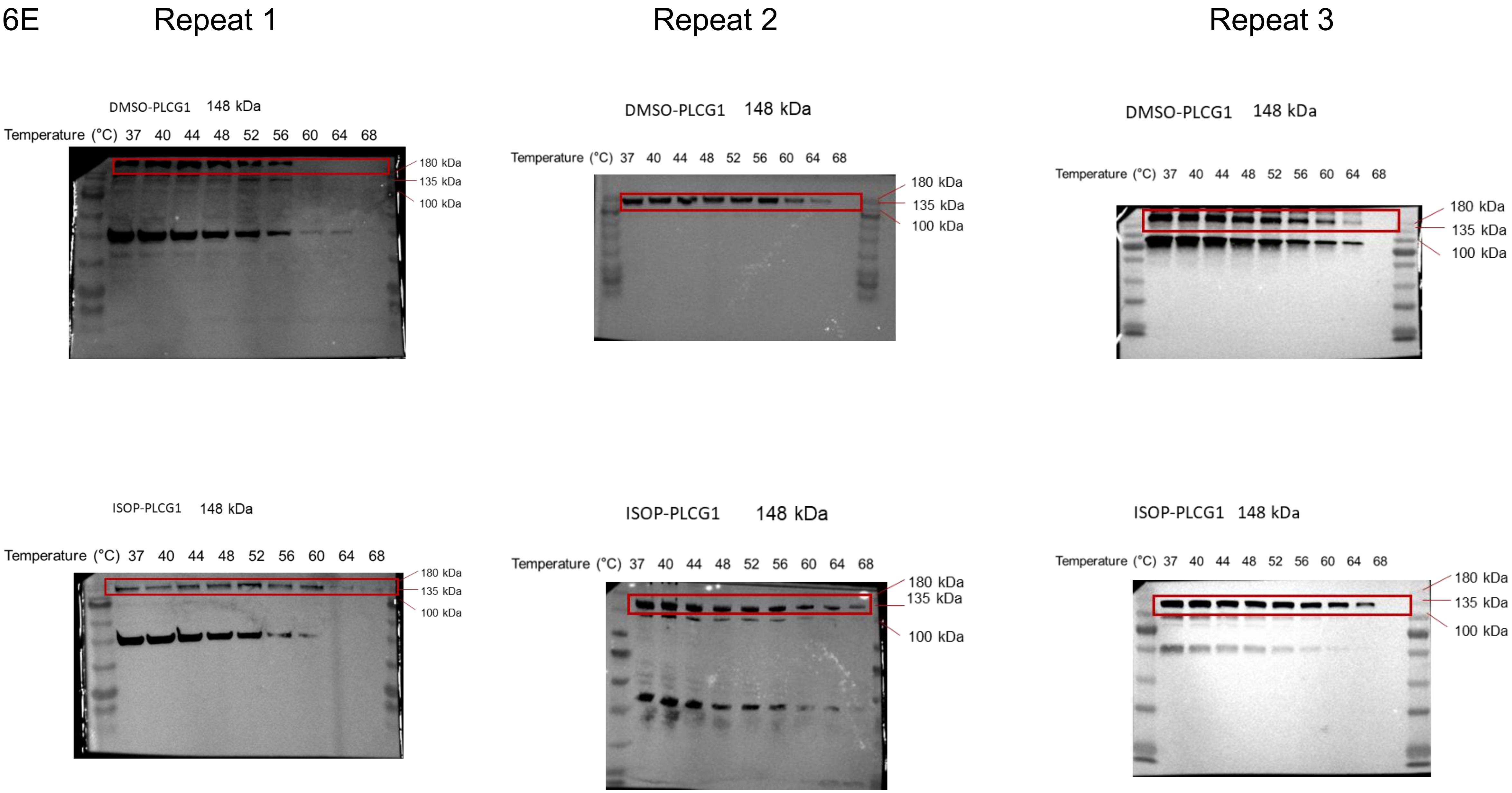
Repeat 1
Repeat 2
Repeat 3
DMSO-PLCG1 148 kDa
Temperature (°C) 37 40 44 48 52 56 60 64 68
180 kDa
135 kDa
100 kDa
DMSO-PLCG1 148 kDa
Temperature (°C) 37 40 44 48 52 56 60 64 68
180 kDa
135 kDa
100 kDa
DMSO-PLCG1 148 kDa
Temperature (°C) 37 40 44 48 52 56 60 64 68
180 kDa
135 kDa
100 kDa
ISOP-PLCG1 148 kDa
Temperature (°C) 37 40 44 48 52 56 60 64 68
180 kDa
135 kDa
100 kDa
ISOP-PLCG1 148 kDa
Temperature (°C) 37 40 44 48 52 56 60 64 68
180 kDa
135 kDa
100 kDa
ISOP-PLCG1 148 kDa
Temperature (°C) 37 40 44 48 52 56 60 64 68
180 kDa
135 kDa
100 kDa

Figure 6G

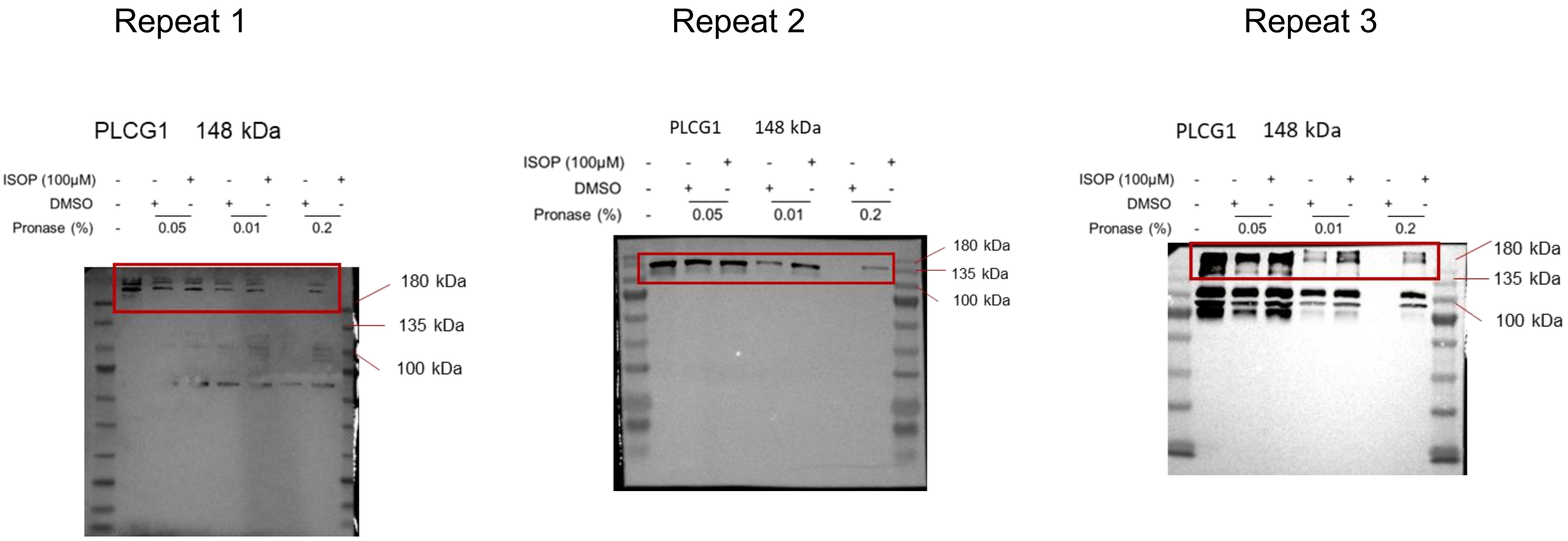

Repeat 1
PLCG1 148 kDa
ISOP (100µM) - - + - + - +
DMSO - + - + - + -
Pronase (%) - 0.05 0.01 0.2
180 kDa
135 kDa
100 kDa
Repeat 2
PLCG1 148 kDa
ISOP (100µM) - - + - + - +
DMSO - + - + - + -
Pronase (%) - 0.05 0.01 0.2
180 kDa
135 kDa
100 kDa
Repeat 3
PLCG1 148 kDa
ISOP (100µM) - - + - + - +
DMSO - + - + - + -
Pronase (%) - 0.05 0.01 0.2
180 kDa
135 kDa
100 kDa

Figure 6I

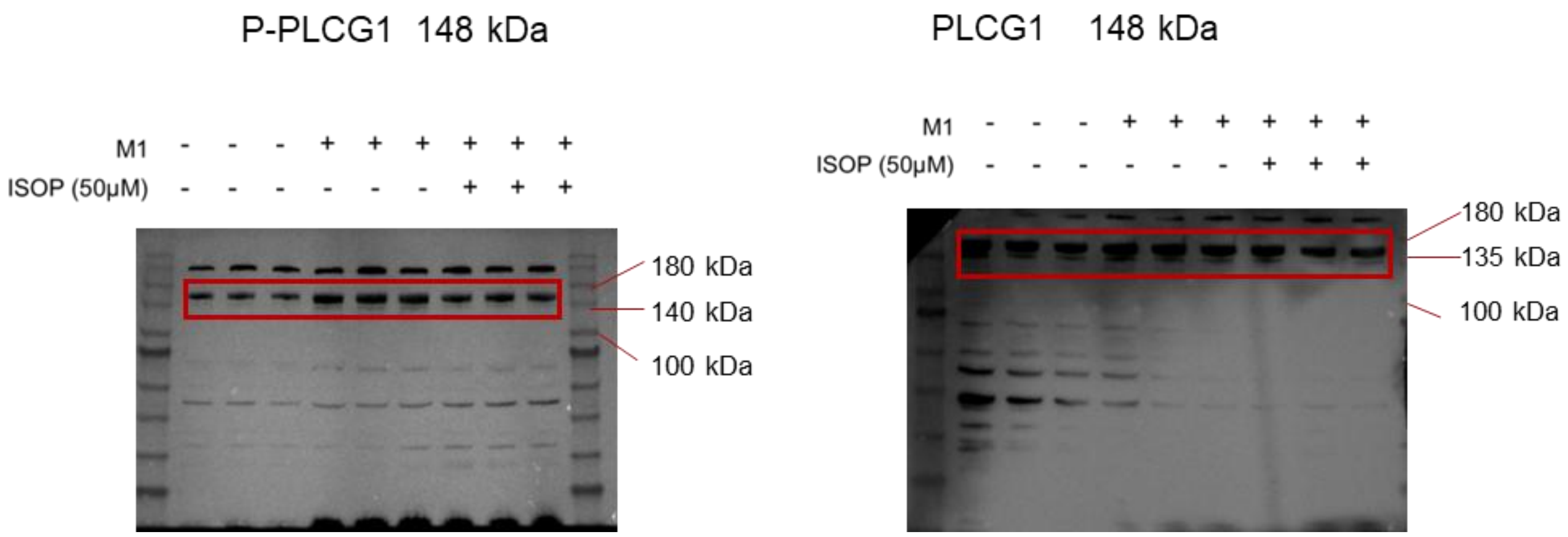
P-PLCG1 148 kDa
M1 - - - + + + + + +
ISOP (50μM) - - - - - - + + +
180 kDa
140 kDa
100 kDa
PLCG1 148 kDa
M1 - - - + + + + + +
ISOP (50μM) - - - - - - + + +
180 kDa
135 kDa
100 kDa

Figure 7B

Repeat 1

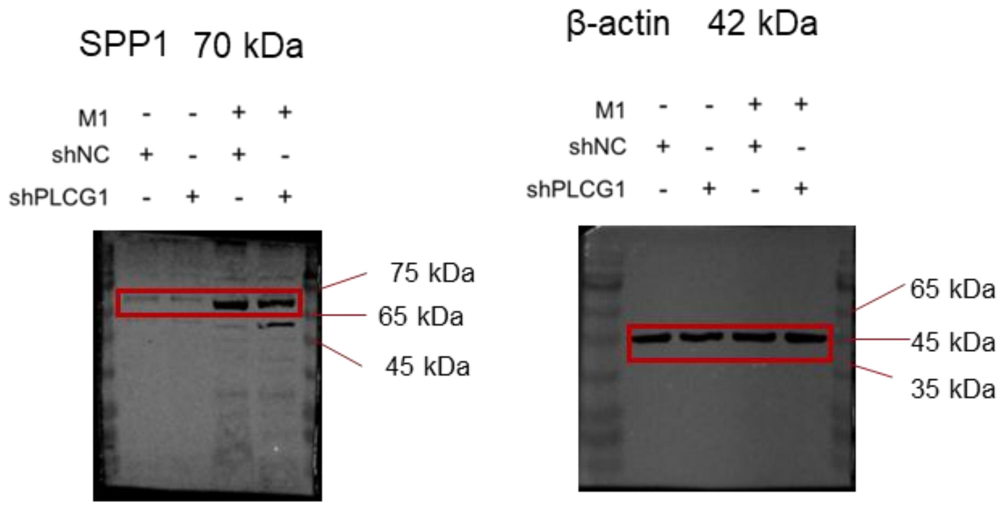

Repeat 2

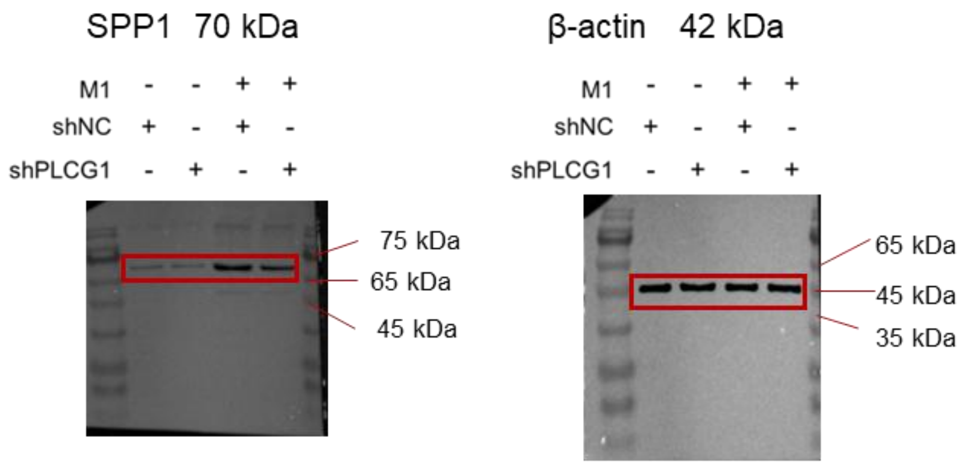

Repeat 3

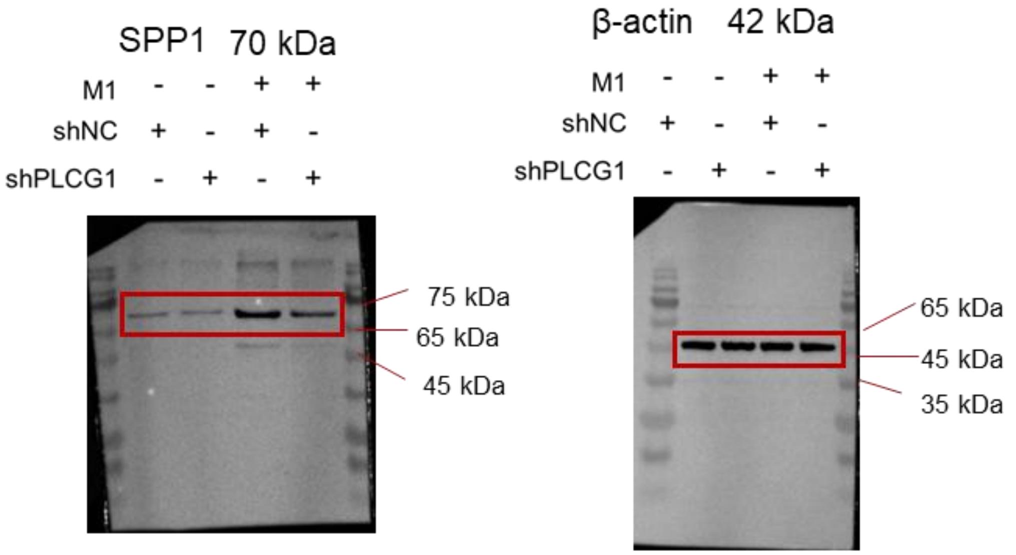

Figure 7D

Repeat 1

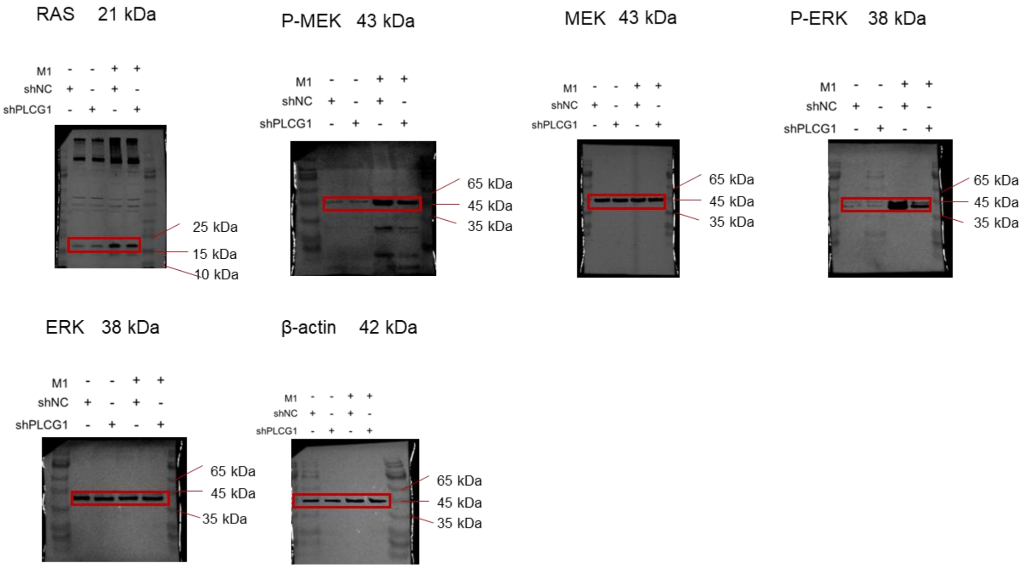

Repeat 2

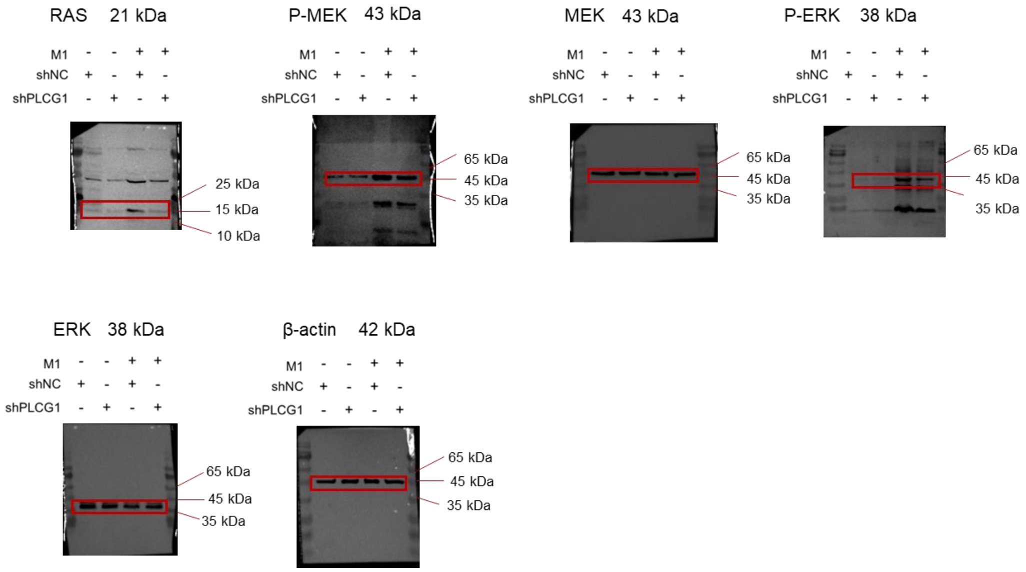

Repeat 3

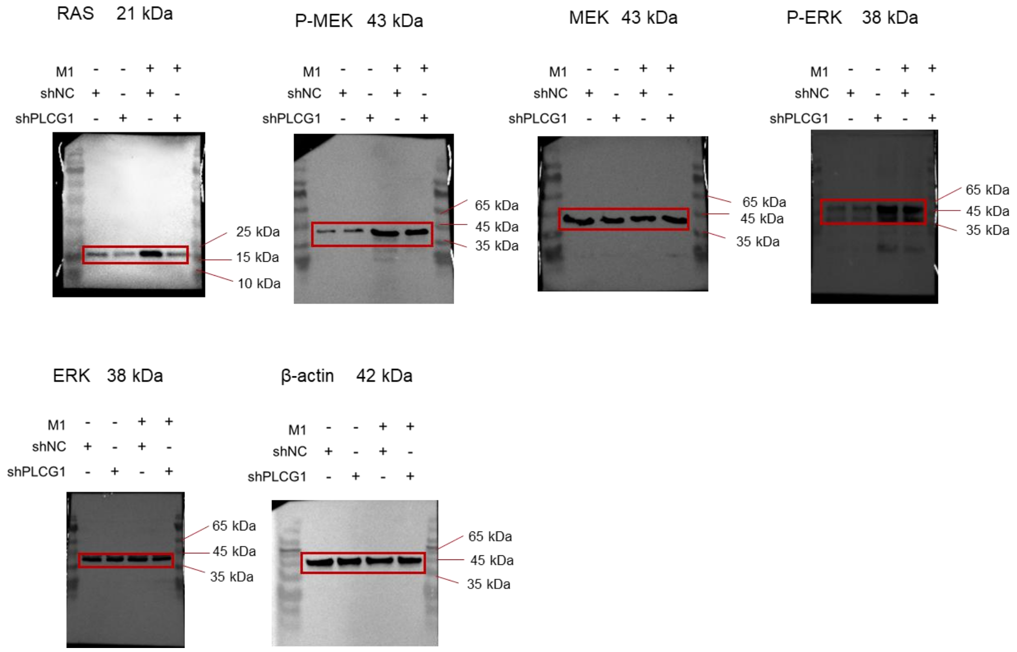

Figure 7I

Repeat 1

SPP1 70 kDa

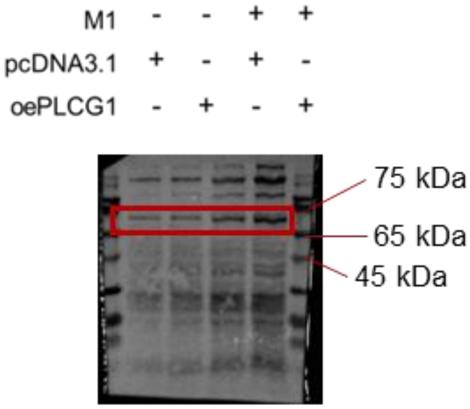

β-actin 42 kDa

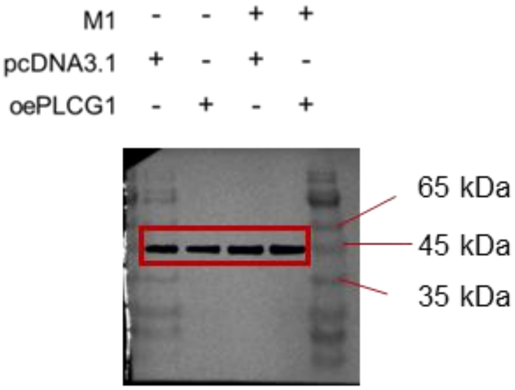

Repeat 2

SPP1 70 kDa

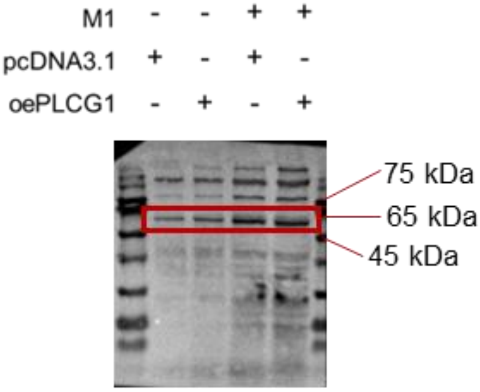

β-actin 42 kDa

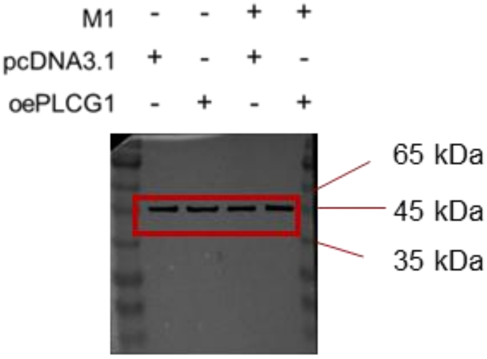

Repeat 3

SPP1 70 kDa

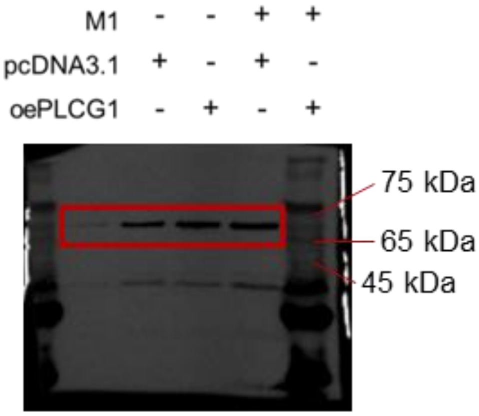

β-actin 42 kDa

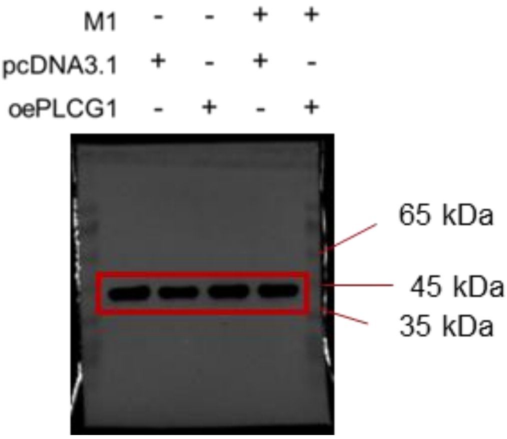

Figure 7K

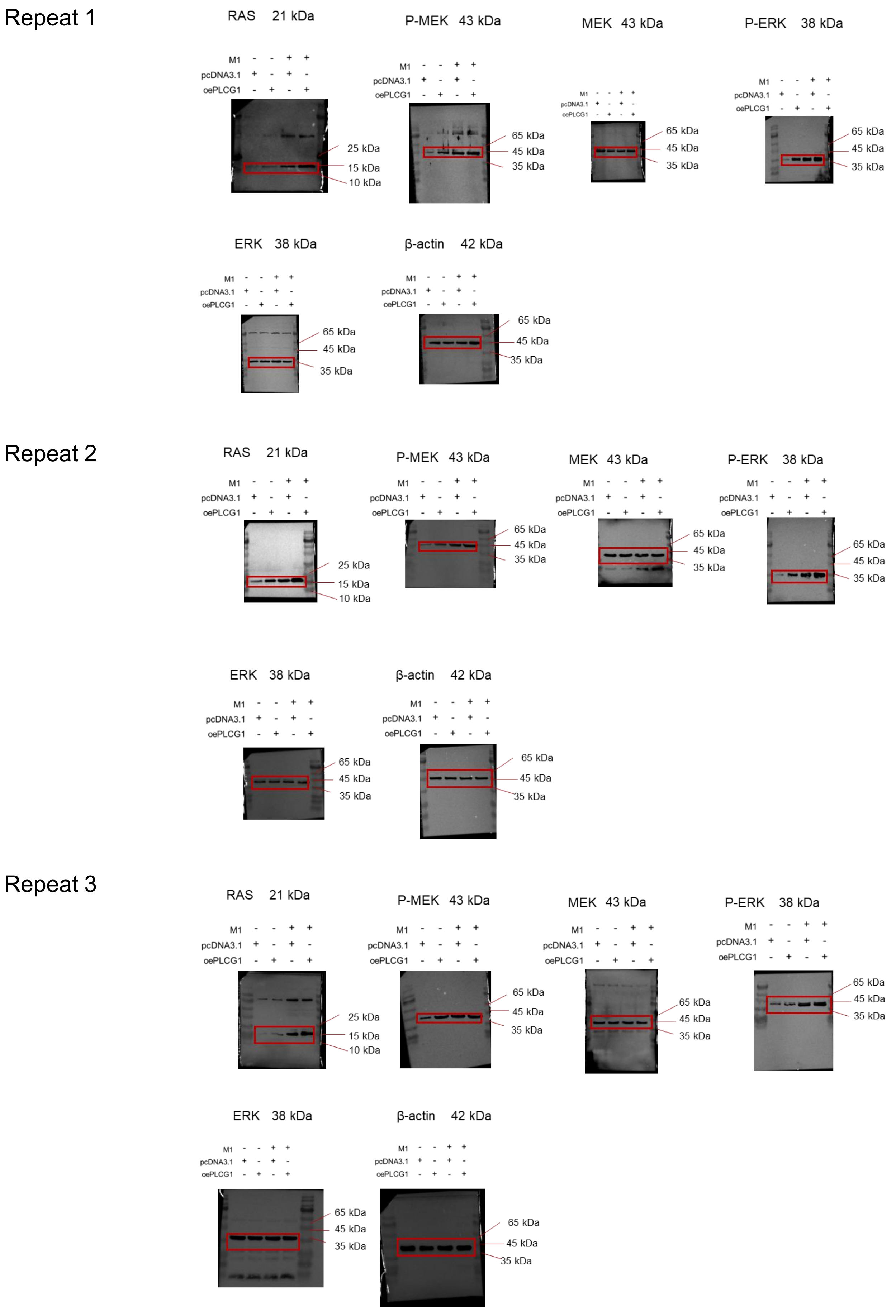
Repeat 1
RAS 21 kDa
M1 - - + +
pcDNA3.1 + - + -
oePLCG1 - + - +
25 kDa
15 kDa
10 kDa
P-MEK 43 kDa
M1 - - + +
pcDNA3.1 + - + -
oePLCG1 - + - +
65 kDa
45 kDa
35 kDa
MEK 43 kDa
M1 - - + +
pcDNA3.1 + - + -
oePLCG1 - + - +
65 kDa
45 kDa
35 kDa
P-ERK 38 kDa
M1 - - + +
pcDNA3.1 + - + -
oePLCG1 - + - +
65 kDa
45 kDa
35 kDa
ERK 38 kDa
M1 - - + +
pcDNA3.1 + - + -
oePLCG1 - + - +
65 kDa
45 kDa
35 kDa
β-actin 42 kDa
M1 - - + +
pcDNA3.1 + - + -
oePLCG1 - + - +
65 kDa
45 kDa
35 kDa
Repeat 2
RAS 21 kDa
M1 - - + +
pcDNA3.1 + - + -
oePLCG1 - + - +
25 kDa
15 kDa
10 kDa
P-MEK 43 kDa
M1 - - + +
pcDNA3.1 + - + -
oePLCG1 - + - +
65 kDa
45 kDa
35 kDa
MEK 43 kDa
M1 - - + +
pcDNA3.1 + - + -
oePLCG1 - + - +
65 kDa
45 kDa
35 kDa
P-ERK 38 kDa
M1 - - + +
pcDNA3.1 + - + -
oePLCG1 - + - +
65 kDa
45 kDa
35 kDa
ERK 38 kDa
M1 - - + +
pcDNA3.1 + - + -
oePLCG1 - + - +
65 kDa
45 kDa
35 kDa
β-actin 42 kDa
M1 - - + +
pcDNA3.1 + - + -
oePLCG1 - + - +
65 kDa
45 kDa
35 kDa
Repeat 3
RAS 21 kDa
M1 - - + +
pcDNA3.1 + - + -
oePLCG1 - + - +
25 kDa
15 kDa
10 kDa
P-MEK 43 kDa
M1 - - + +
pcDNA3.1 + - + -
oePLCG1 - + - +
65 kDa
45 kDa
35 kDa
MEK 43 kDa
M1 - - + +
pcDNA3.1 + - + -
oePLCG1 - + - +
65 kDa
45 kDa
35 kDa
P-ERK 38 kDa
M1 - - + +
pcDNA3.1 + - + -
oePLCG1 - + - +
65 kDa
45 kDa
35 kDa
ERK 38 kDa
M1 - - + +
pcDNA3.1 + - + -
oePLCG1 - + - +
65 kDa
45 kDa
35 kDa
β-actin 42 kDa
M1 - - + +
pcDNA3.1 + - + -
oePLCG1 - + - +
65 kDa
45 kDa
35 kDa

Figure 8B

Repeat 1

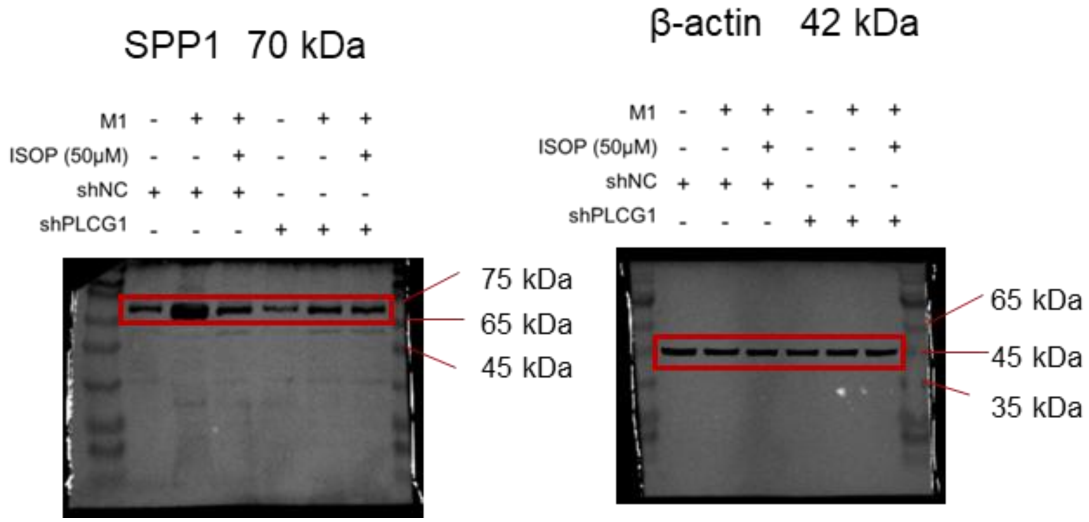

Repeat 2

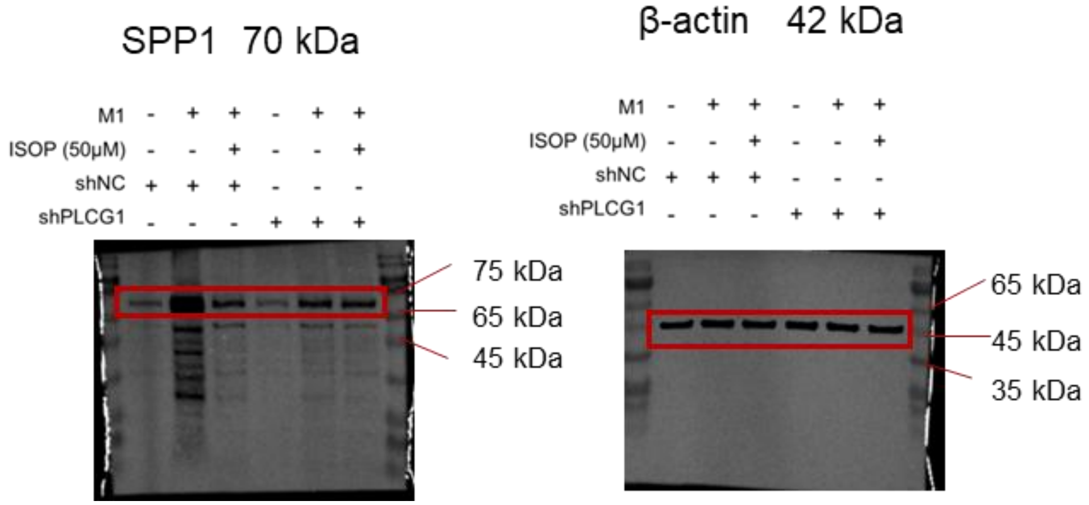

Repeat 3

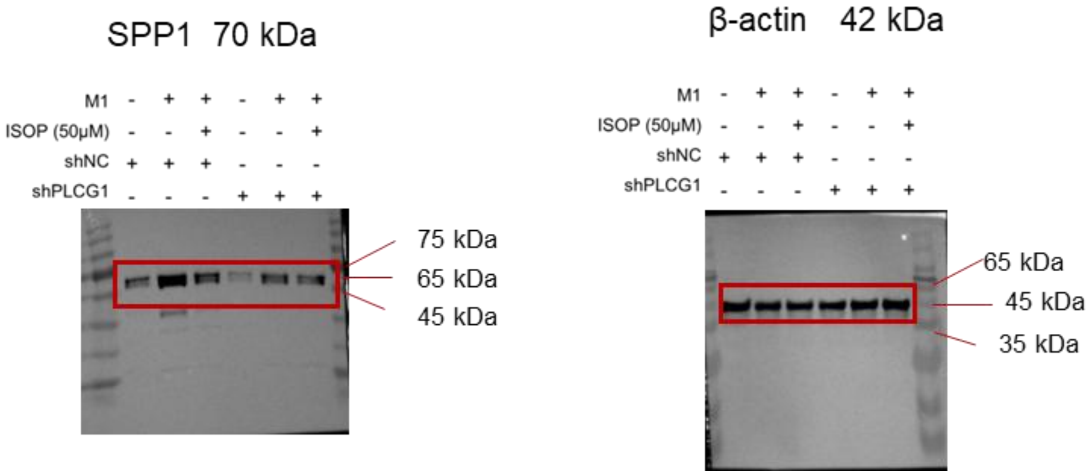

Figure 8D

Repeat 1

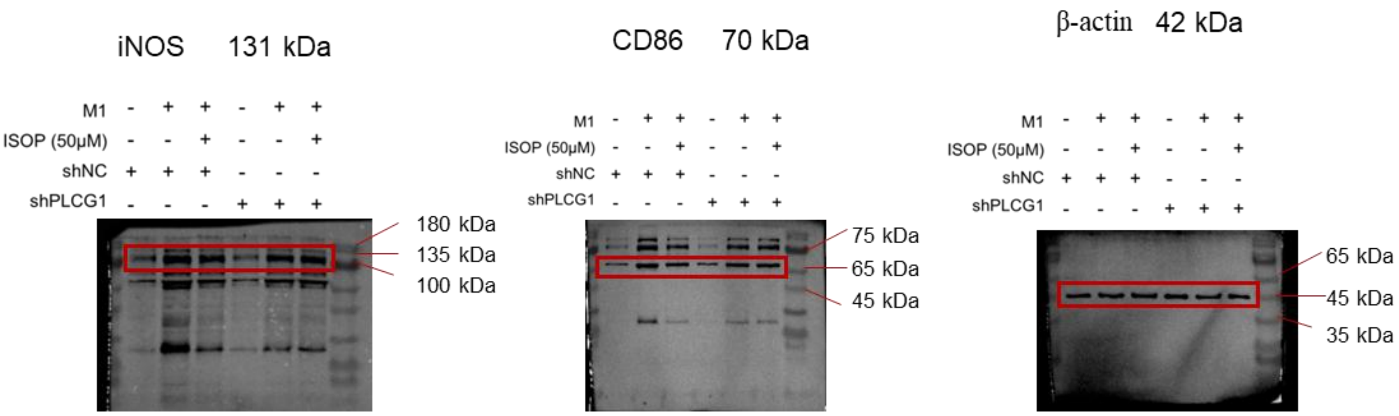

Repeat 2

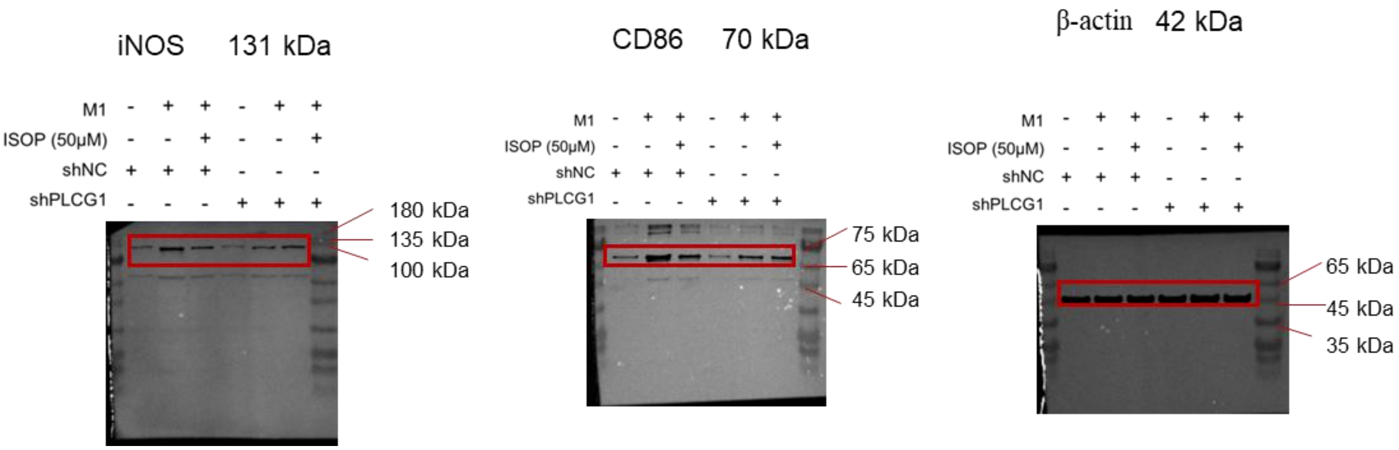

Repeat 3

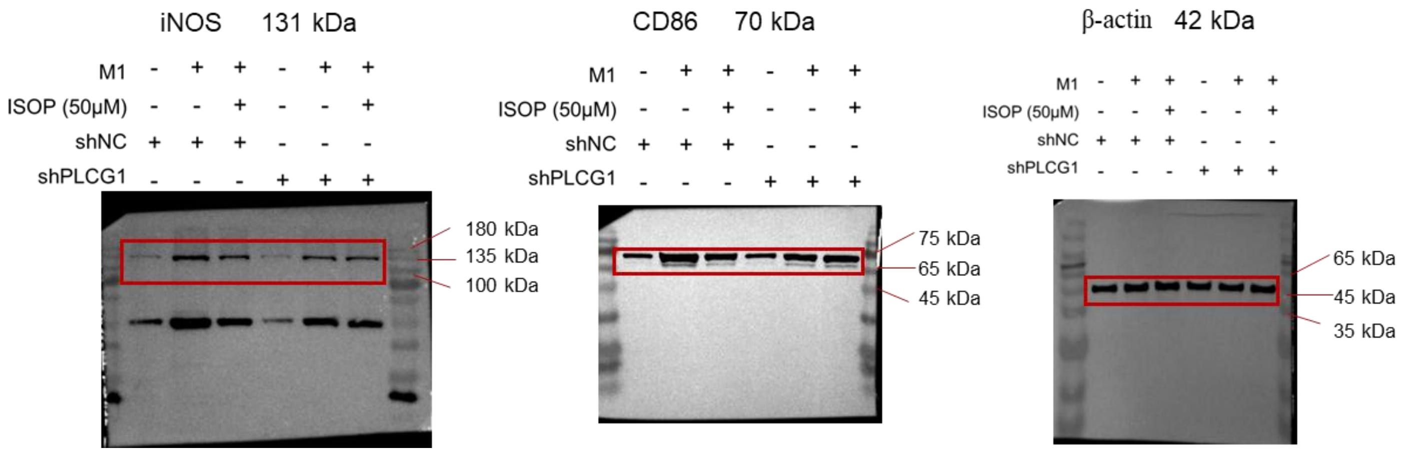

Figure 8M

Repeat 1

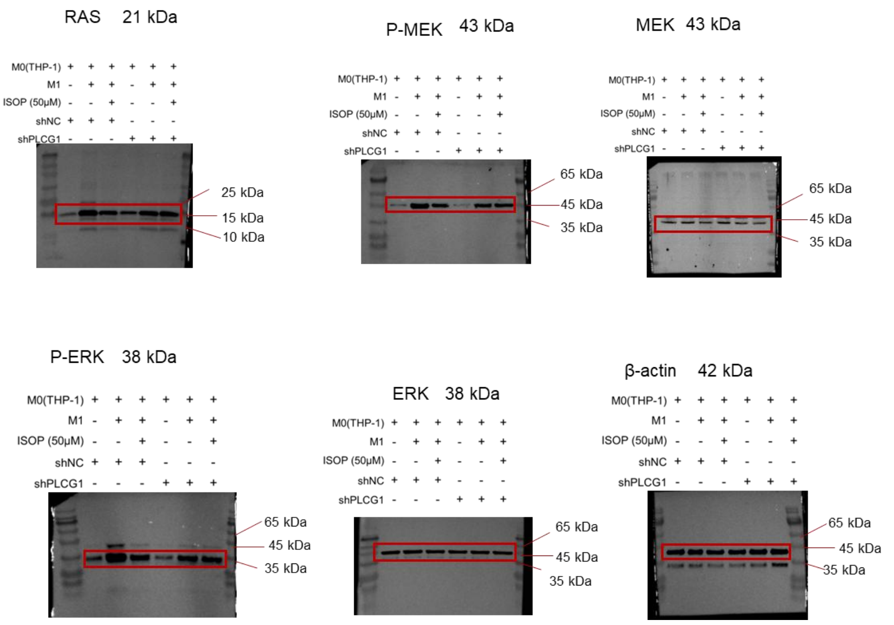

Repeat 2

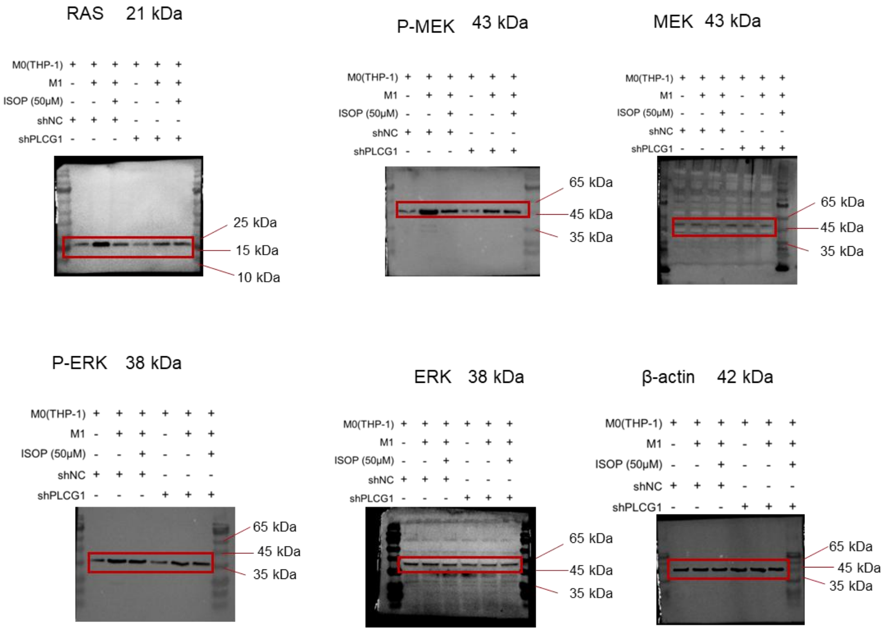

Repeat 3

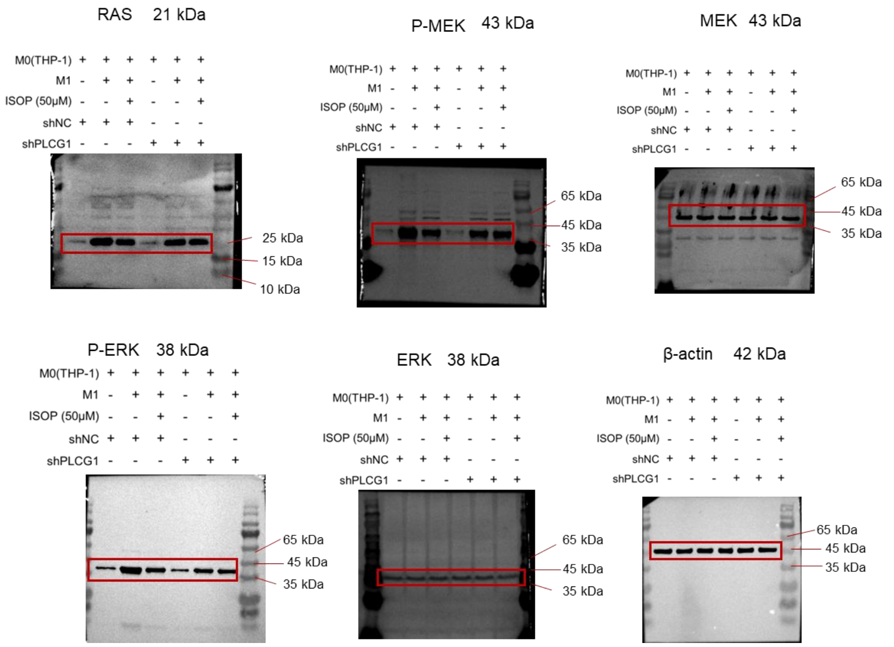

Figure 9D

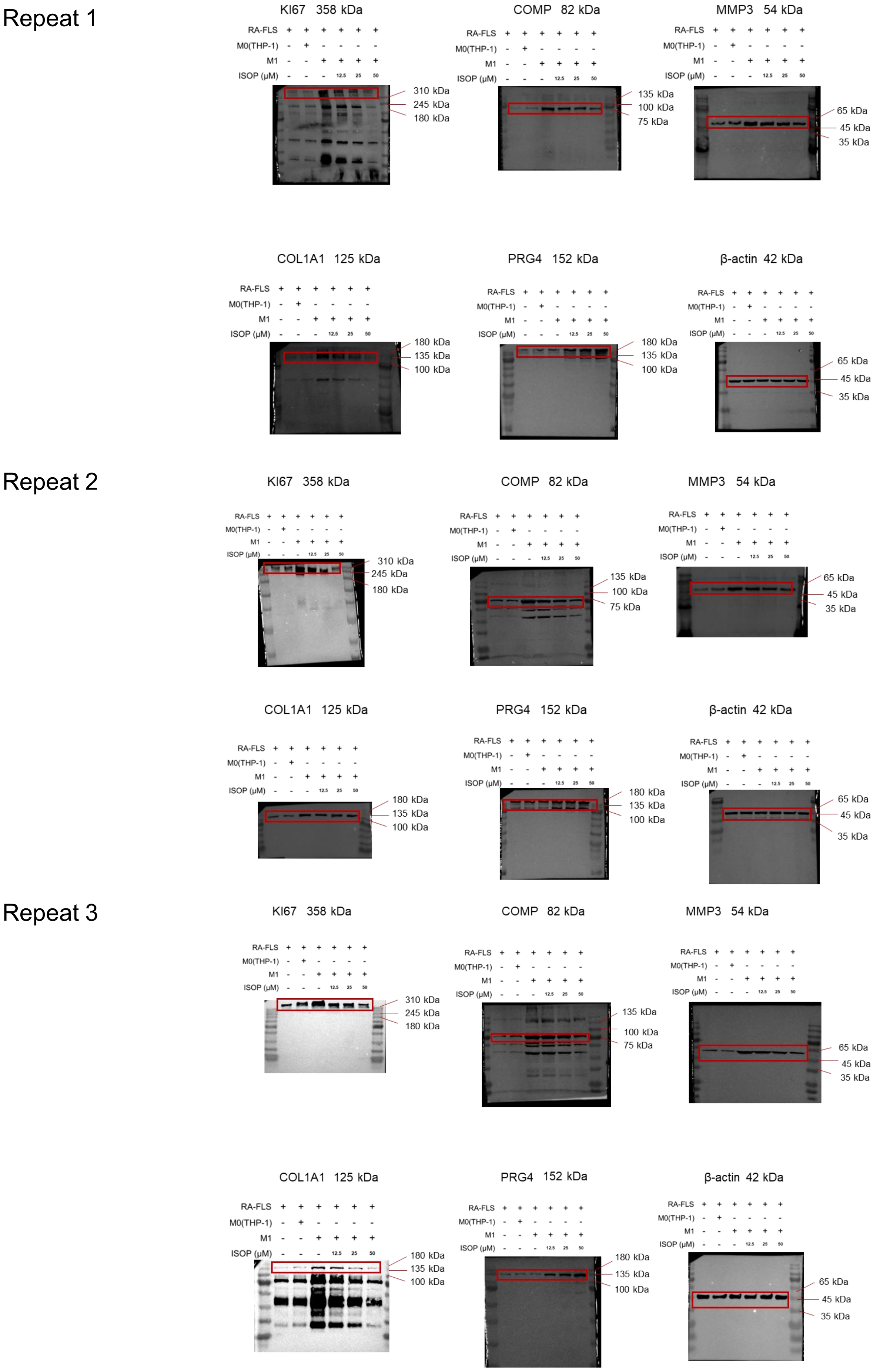
Repeat 1
KI67 358 kDa
COMP 82 kDa
MMP3 54 kDa
COL1A1 125 kDa
PRG4 152 kDa
β-actin 42 kDa
Repeat 2
KI67 358 kDa
COMP 82 kDa
MMP3 54 kDa
COL1A1 125 kDa
PRG4 152 kDa
β-actin 42 kDa
Repeat 3
KI67 358 kDa
COMP 82 kDa
MMP3 54 kDa
COL1A1 125 kDa
PRG4 152 kDa
β-actin 42 kDa
RA-FLS + + + + + +
M0(THP-1) - + - - - -
M1 - - + + + +
ISOP (μM) - - - 12.5 25 50
310 kDa
245 kDa
180 kDa
135 kDa
100 kDa
75 kDa
65 kDa
45 kDa
35 kDa

Figure 9L

Repeat 1

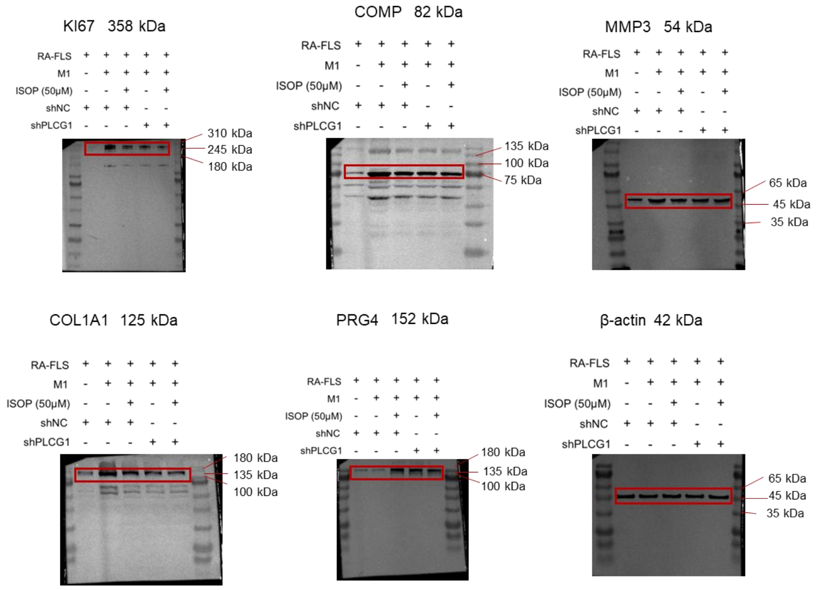

Repeat 2

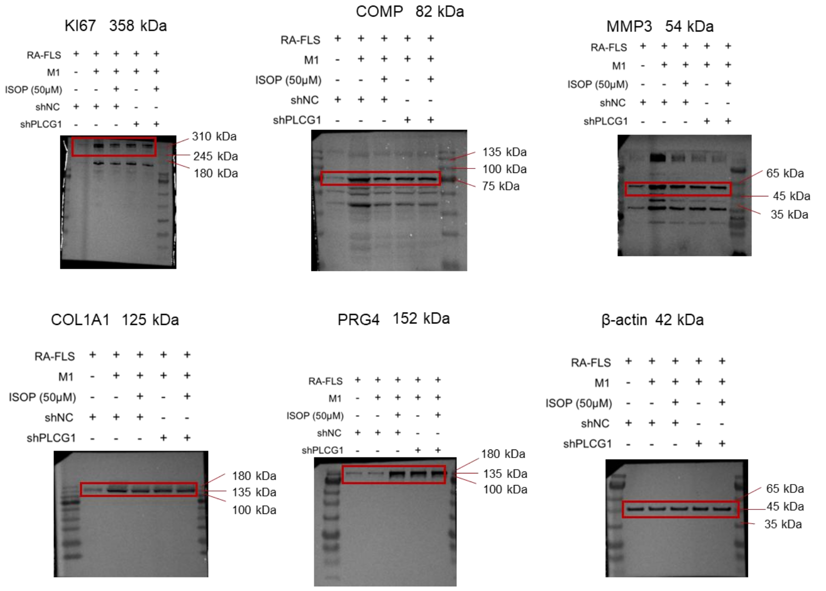

Repeat 3

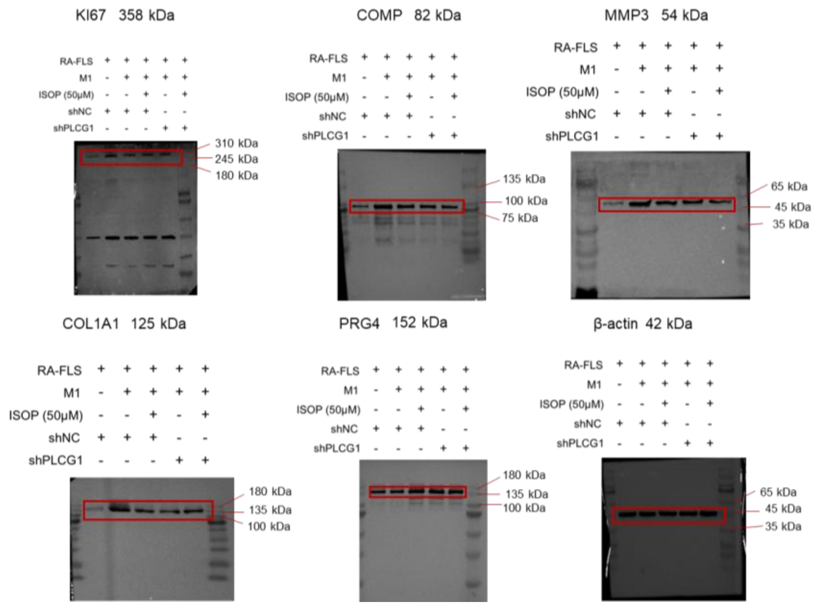

Supplement: Supplementary file 2 — Additional file 2. [file 11658_2026_918_MOESM2_ESM.pdf]
